# Structural and biochemical analysis of a novel atypically split intein reveals a conserved histidine specific to cysteine-less inteins†

**DOI:** 10.1039/d3sc01200j

**Published:** 2023-04-24

**Authors:** Tim Pasch, Alexander Schröder, Sabrina Kattelmann, Miriam Eisenstein, Shmuel Pietrokovski, Daniel Kümmel, Henning D. Mootz

**Affiliations:** a Institute of Biochemistry, University of Münster Corrensstr. 36 48149 Münster Germany Henning.Mootz@uni-muenster.de; b Department of Molecular Genetics, Weizmann Institute of Science Rehovot 76100 Israel shmuel.pietrokovski@weizmann.ac.il

## Abstract

Protein *trans*-splicing mediated by a split intein reconstitutes a protein backbone from two parts. This virtually traceless autoprocessive reaction provides the basis for numerous protein engineering applications. Protein splicing typically proceeds through two thioester or oxyester intermediates involving the side chains of cysteine or serine/threonine residues. A cysteine-less split intein has recently attracted particular interest as it can splice under oxidizing conditions and is orthogonal to disulfide or thiol bioconjugation chemistries. Here, we report the split PolB16 OarG intein, a second such cysteine-independent intein. As a unique trait, it is atypically split with a short intein-N precursor fragment of only 15 amino acids, the shortest characterized to date, which was chemically synthesized to enable protein semi-synthesis. By rational engineering we obtained a high-yielding, improved split intein mutant. Structural and mutational analysis revealed the dispensability of the usually crucial conserved motif N3 (block B) histidine as an obvious peculiar property. Unexpectedly, we identified a previously unnoticed histidine in hydrogen-bond forming distance to the catalytic serine 1 as critical for splicing. This histidine has been overlooked so far in multiple sequence alignments and is highly conserved only in cysteine-independent inteins as a part of a newly discovered motif NX. The motif NX histidine is thus likely of general importance to the specialized environment in the active site required in this intein subgroup. Together, our study advances the toolbox as well as the structural and mechanistic understanding of cysteine-less inteins.

## Abstract 2

Cysteine-less split inteins are very useful, but rare tools for protein engineering. Investigation of the novel PolB16 intein revealed a previously overlooked histidine as a conserved part for the serine-dependent mechanism.

## Graphical abstract

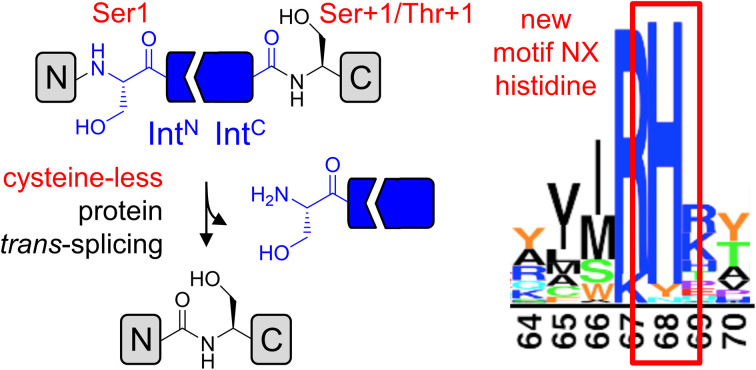

## Introduction

**Fig. 1:**
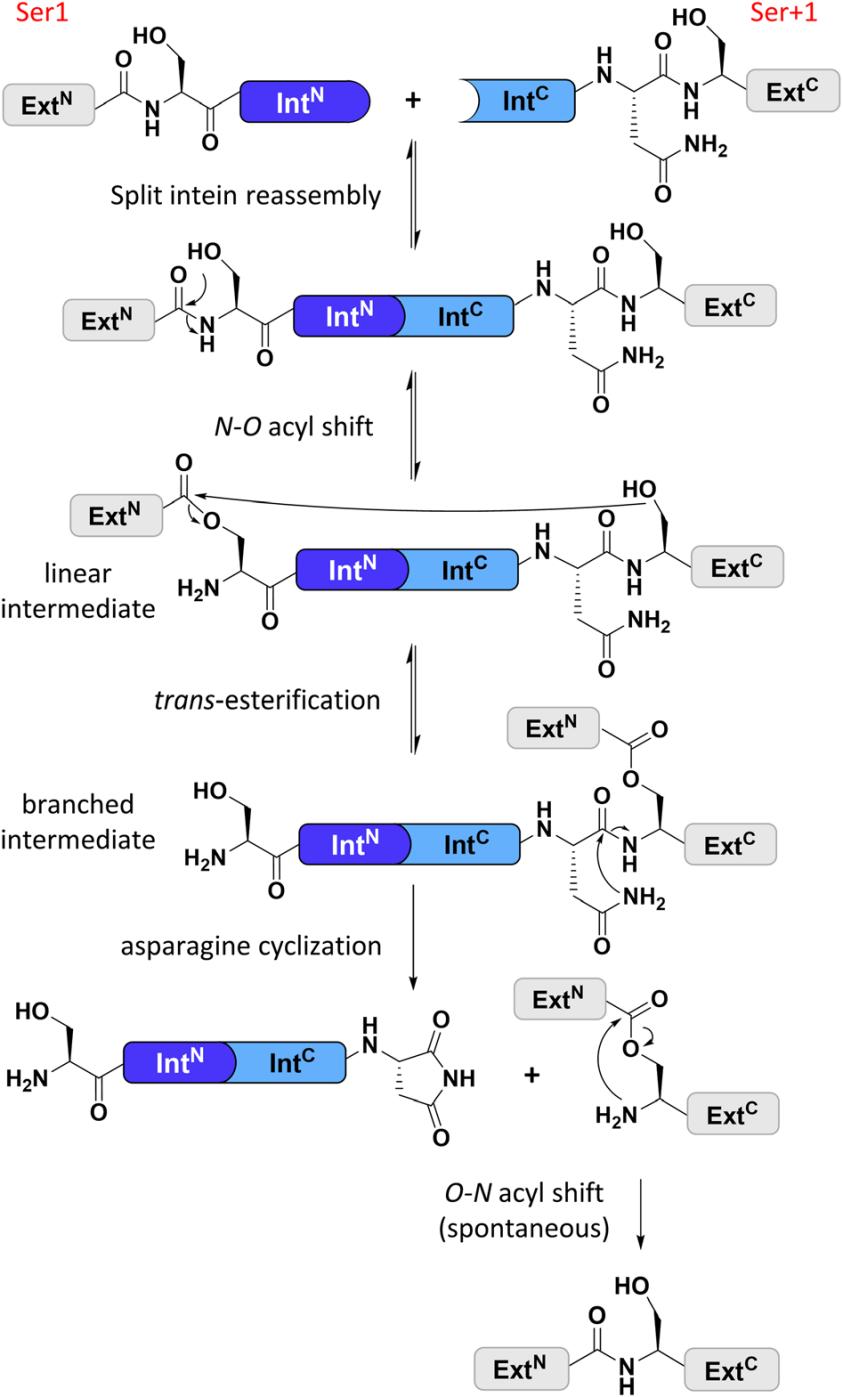
General protein splice mechanism of a cysteine-less split intein. Note that such inteins operate with Ser1 and Ser+1 (Thr+1) residues at the two splice junctions to form the linear and branched ester intermediates. Ext^N^, Ext^C^ = N- and C-terminal exteins, respectively.

Split inteins reconstitute a host protein from two separate pieces to a single polypeptide chain. This peptide ligation reaction, termed protein *trans*-splicing, proceeds through one or two covalent thioester or ester intermediates on conserved cysteine, serine, or threonine residues and joins the intein flanks with a peptide bond (see Fig. 1). Because inteins excise themselves during the reaction, the ligation is virtually traceless, with the remaining +1 catalytic residue at the ligation site being part of the host protein (Fig. 1).^1–3^ Transplanting split inteins into a heterologous sequence context of choice enables a plethora of protein engineering applications.^2–7^ Therefore, functionally robust split inteins are being urgently sought after. The split inteins' specific properties and sequence features can be critical for the particular protein ligation challenge. For example, short split N- or C-terminal intein fragments (Int^N^ or Int^C^) are amenable to solid-phase peptide synthesis and enable semi-synthetic protein *trans*-splicing.^8–11^

Cysteine-less inteins are inteins lacking cysteine residues, first and foremost at their 1 and +1 positions which are involved in the formation of linear and branched intermediates, with the +1 position formally representing the first C-extein residue (Fig. 1). Cysteine-less inteins can catalyze the protein *trans*-splicing reaction under oxidizing conditions and without adding reducing agents. Under these conditions, the more abundant cysteine-dependent counterparts can be impaired in their efficiency due to partial or quantitative oxidation of their cysteine thiol moieties. This is particularly critical for split intein precursors that have been separately produced. Thus, cysteine-less split inteins significantly expand our toolbox for protein engineering applications, for example, to preserve critical disulfide bridges in proteins of interest (POI)^12^ or for novel combinations with thiol-dependent bioconjugation reactions.^12–15^ However, cysteine-less split inteins are very rare. The Aes123 PolB1 intein and its engineered CL variant provide the only such reported and characterized examples with useful activity.^12^ The previously reported Neq Pol intein was the first discovered naturally split cysteine-less intein,^16^ however, as a thermophilic protein it required temperatures of at least 50–70 °C to show reasonable activity.^16^ The other published example of a cysteine-less split intein is the Psp-GBD Pol intein, which was artificially generated from its contiguous, *cis*-splicing parent intein. Likely because of this engineering, the separate split intein precursors did not assemble properly and required a denaturation–renaturation cycle for refolding into the active form.^15,17^ The high temperature and the required refolding preclude the Neq Pol and the Psp-GBD Pol inteins, respectively, from a practical utility for most applications.

Identifying novel cysteine-less split inteins requires locating the active site serine or threonine residues at the 1 and +1 positions. However, it is unclear how the nucleophilic side chains at those positions are selected in intein evolution. In a survey of all *cis* and *trans*-splicing inteins of the intein database, only 10% exhibited only serine or threonine residues at both these positions.^18^ The +1 position appears more permissive for site-directed mutagenesis than the 1 position, as a couple of examples of C+1S substitutions with retained splicing activity were reported.^19,20^ In contrast, the Cys1 residue appears much more refractory to changing the side chain thiol to an alcohol moiety, as for several inteins a C1S mutation was shown to result in an inactive intein.^18,20^ No attempt to convert a cysteine-dependent intein into a cysteine-less intein useful for preparative purposes has been successful so far. Obviously, inteins exhibit a high level of specialization around the active site, with a Ser1 residue requiring a dedicated activation mechanism that the active site of a Cys1 intein cannot provide.

Artificially splitting continuous *cis* inteins is another route to obtain split inteins. Indeed, several *cis* inteins without cysteine at the 1 and +1 positions are known, either with or without cysteines at other non-conserved positions. However, folding and assembly problems observed for the above-mentioned artificially split Psp-GBD Pol intein^17^ underline the potential drawbacks of this strategy and have also been observed for several other artificially split inteins.^21–23^ Therefore, identifying naturally occurring split inteins is considered the more attractive route to well-behaved variants^19,24,25^ and thus also the most promising path to powerful novel cysteine-less split inteins.

Here, we report the novel and atypically split PolB16 OarG intein and its biochemical and structural characterization. By replacement of two non-conserved cysteines, it could be mutated into a completely cysteine-less variant and further engineered for efficient splicing properties. Strikingly, we uncovered a new conserved and indispensable histidine residue, while the typically crucial motif N3 histidine is superfluous in the PolB16 OarG intein. The region of this newly identified histidine is conserved and present in other cysteine-less inteins, and forms the first signature trait specific for them, apart from their splice junctions serine or threonine residues. The PolB16 OarG intein utilizes a novel mechanism for activating the Ser1 residue in the protein-splicing pathway.

## Results and discussion

### Identification of the split PolB16 OarG intein and its utility for cysteine-less protein *trans*-splicing applications

**Fig. 2:**
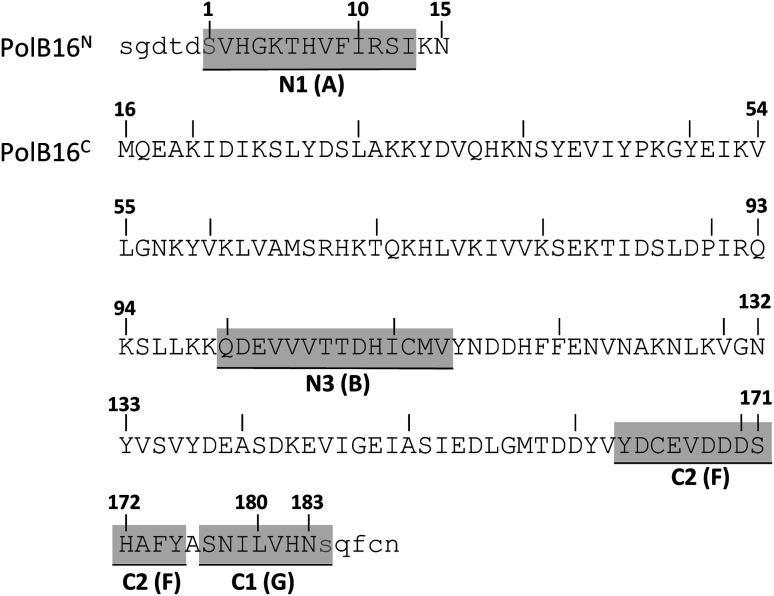
Amino acid sequence of the split PolB16 intein. Shown are the Int^N^ (PolB16^N^) and Int^C^ (PolB16^C^) precursors with 5 extein residues each (in lower cases). Highlighted are the conserved motifs (grey) with the older block nomenclature in brackets, as well as the two catalytic serines at positions 1 and +1 (blue).

We identified the split PolB16 OarG intein, from now on also simply termed PolB16 intein, in sequence data from a sheep gut metagenome.^26^ The intein genes are inserted in a gene for a putative PolB-type DNA-polymerase. Naturally occurring split inteins typically exhibit a longer Int^N^ of about 100 amino acids (aa) and a shorter Int^C^ of about 35–40 aa. However, atypically split inteins with a short Int^N^ fragment and correspondingly longer Int^C^ have recently been identified.^20,27,28^ The PolB16 intein carries an atypical split position close to the N-terminal end of the encoded split intein domain to give Int^N^ and Int^C^ fragments of 15 and 168 aa (Fig. 2), respectively. Most notably, both residues at the 1 and +1 positions are serine. Furthermore, the intein overall exhibits the typical conserved motifs of a class 1 intein^29^ (Fig. 2) that represent conserved residues across inteins, of which some are involved in catalyzing the steps of the protein splicing pathway. This analysis suggested the PolB16 intein splices through linear and branched oxoester intermediates (Fig. 1). The Int^C^ fragment contains two cysteines within motifs N3 and C2,^30^ (previously also referred to as motifs B and F)^31^ however, at non-essential and only moderately conserved positions that usually harbor hydrophobic residues.

**Fig. 3:**
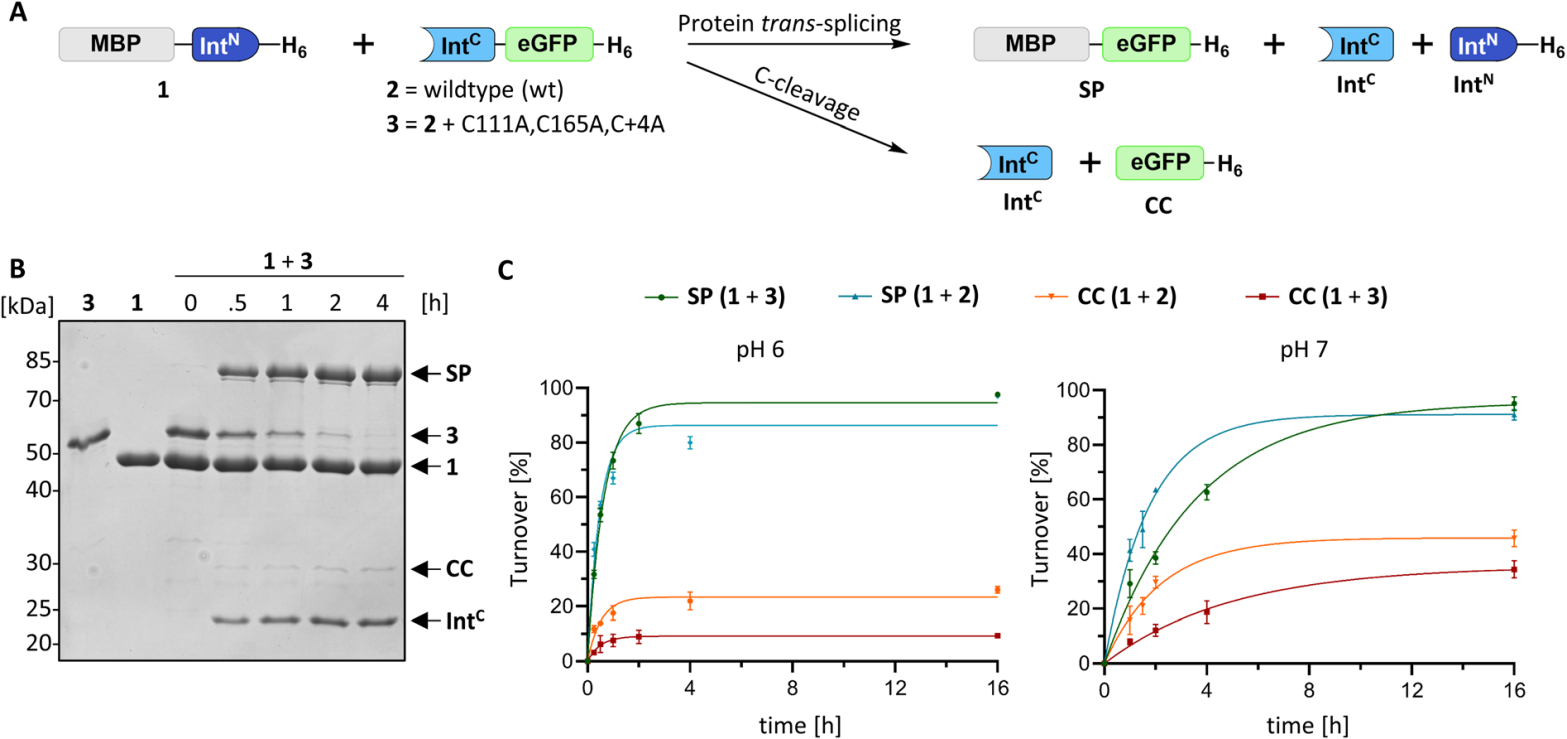
Protein *trans*-splicing of the PolB16 intein and its engineered cysteine-less mutant. (A) Scheme of the reactions. (B) Reactions were performed with the Int^N^ (10 μM) and Int^C^ (5 μM) precursors at 25 °C and pH 6.0. Shown is a representative coomassie-stained SDS-PAGE gel analysis with the Int^C^ precursor mutant devoid of any cysteines. (C) Densitometric analyses of the reactions under the same conditions as in (B), but at the indicated pH values (*n* = 3 for each reaction; error bars represent standard deviations). SP = splice product. CC = C-terminal cleavage product.

For a biochemical characterization, we produced recombinant constructs of the PolB16 intein fragments in *E. coli*. Each fragment contained 5 native extein residues, fused to maltose-binding protein (MBP) and enhanced green fluorescent protein (eGFP) to give MBP-Int^N^-H_6_ (1) and Int^C^-eGFP-H_6_ (2), respectively. Upon mixing these purified model proteins, we observed virtually complete turnover of the Int^C^ precursor (2) at pH 7.0 and 25 °C (Fig. 3A and S1B†). We made similar findings for the mutated Int^C^ precursor Int^C^(C111A, C165A)-eGFP-H_6_) (3), suggesting that both non-conserved cysteines can be replaced without impairing intein activity (Fig. 3). In this fully cysteine-less Int^C^ precursor the cysteine in the +4 position of the flanking extein region was also replaced with alanine.

We varied temperature, salt concentration and pH conditions to reveal the optimal activity of the cysteine-less PolB16 split intein in terms of efficiency at pH 6.0 and 25 °C (Fig. S2†). Fig. 3B shows that the splice product MBP-eGFP-H_6_ was formed with 89% yield under these conditions. As a by-product of C-terminal cleavage, eGFP-H_6_ was found with 9.5% yield (Fig. 3C). Importantly, these protein *trans*-splicing assays led to the same results in the absence and presence of reducing agents like DTT and TCEP. At pH 7 we found the wild-type split PolB16 intein (1 + 2) to be twice as fast as its cysteine-less variant (1 + 3) with a reaction constant of 0.16 ± 0.02 × 10^−3^ s^−1^ compared to 0.77 ± 0.01 × 10^−4^ s^−1^, respectively, assuming pseudo-first-order conditions. At pH 6 the rates increase by a factor of 4–6-fold with reaction constants of 0.57 ± 0.05 × 10^−3^ s^−1^*vs.* 0.43 ± 0.03 × 10^−3^ s^−1^. The corresponding half-life times are *t*_½_ = 73 min and 150 min at pH 7, as well as 20 min and 27 min at pH 6, respectively. The improved rates and splice products yields observed at pH 6 compared to pH 7 likely stem from the beneficial effect of the lower pH to form the linear intermediate in equilibrium with the peptide bond (Fig. 1), mediated by protonation of the liberated α-amino group of Ser1.^32^

**Fig. 4:**
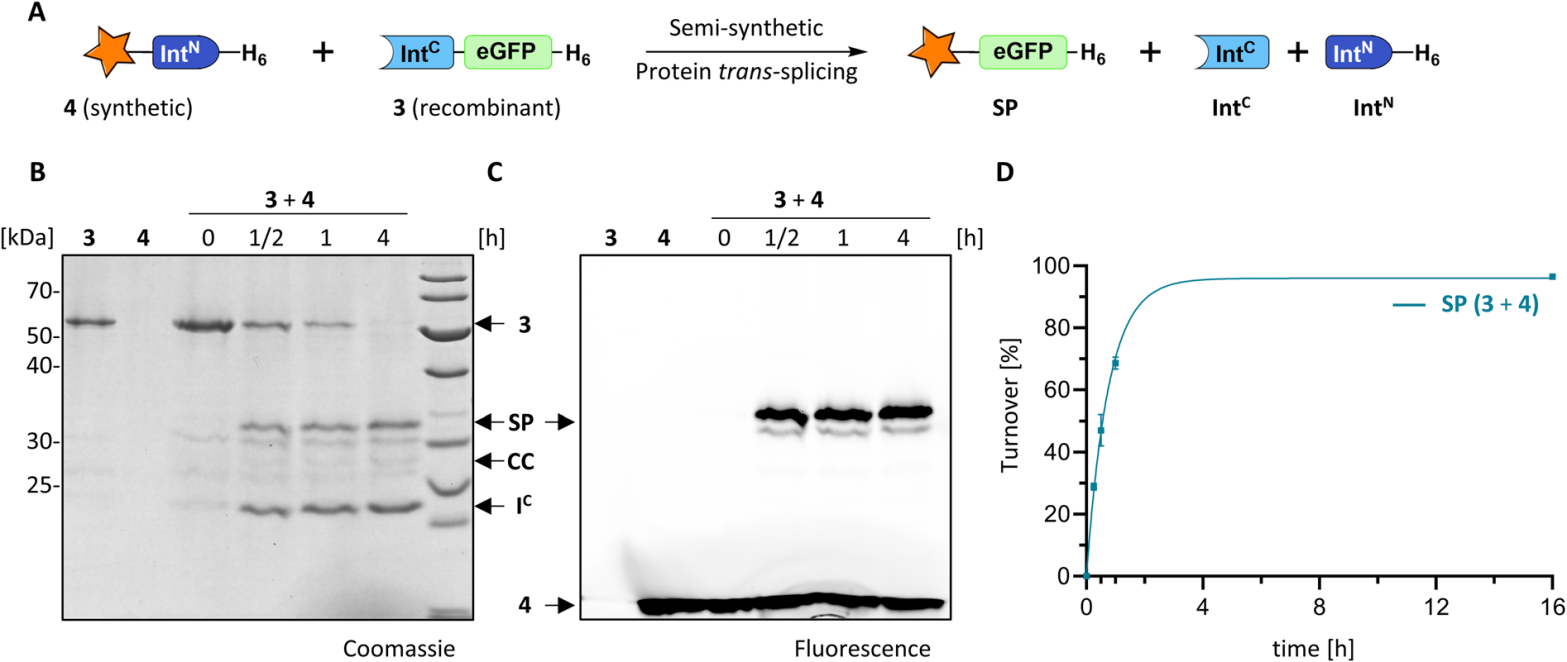
Semi-synthetic protein *trans*-splicing with the PolB16 intein. (A) Scheme of the reaction. Synthetic CF-SGDTD-Int^N^ (4) was prepared by SPPS. (B and C) SDS-PAGE analysis using coomassie-staining or fluorescence, respectively, of the reaction between 4 (15 μM) and 3 (5 μM) at 25 °C and pH 6. (D) Densitometric analysis of the reaction (*n* = 4; error bars represent standard deviations) shown in (B). SP = splice product.

We explored the rare and extremely short Int^N^ fragment of the PolB16 intein and prepared an Int^N^ precursor by solid-phase peptide synthesis (SPPS) with 5(6)-carboxyfluorescein (CF) as a synthetic fluorophore in a short N-extein (CF-SGDTD-Int^N^ construct 4; Fig. S3†). Fig. 4 shows that upon incubation with the Int^C^ precursor 3 virtually quantitative formation of the semi-synthetic splicing product was obtained. The rate of 0.38 ± 0.01 × 10^−3^ s^−1^ was comparable to the fully recombinant intein.

As PolB16 intein is only the second characterized cysteine-less split intein with ambient temperature activity, we also demonstrated that it is orthogonal to the previously described Aes123/CL intein (Fig. S4†),^12^ thus enabling possibilities for dual-splicing applications. Together, these findings establish the engineered, cysteine-less PolB16 intein as a valuable addition to the protein chemistry toolbox.

### The typically critical motif N3 histidine is dispensable in the PolB16 intein

**Fig. 5:**
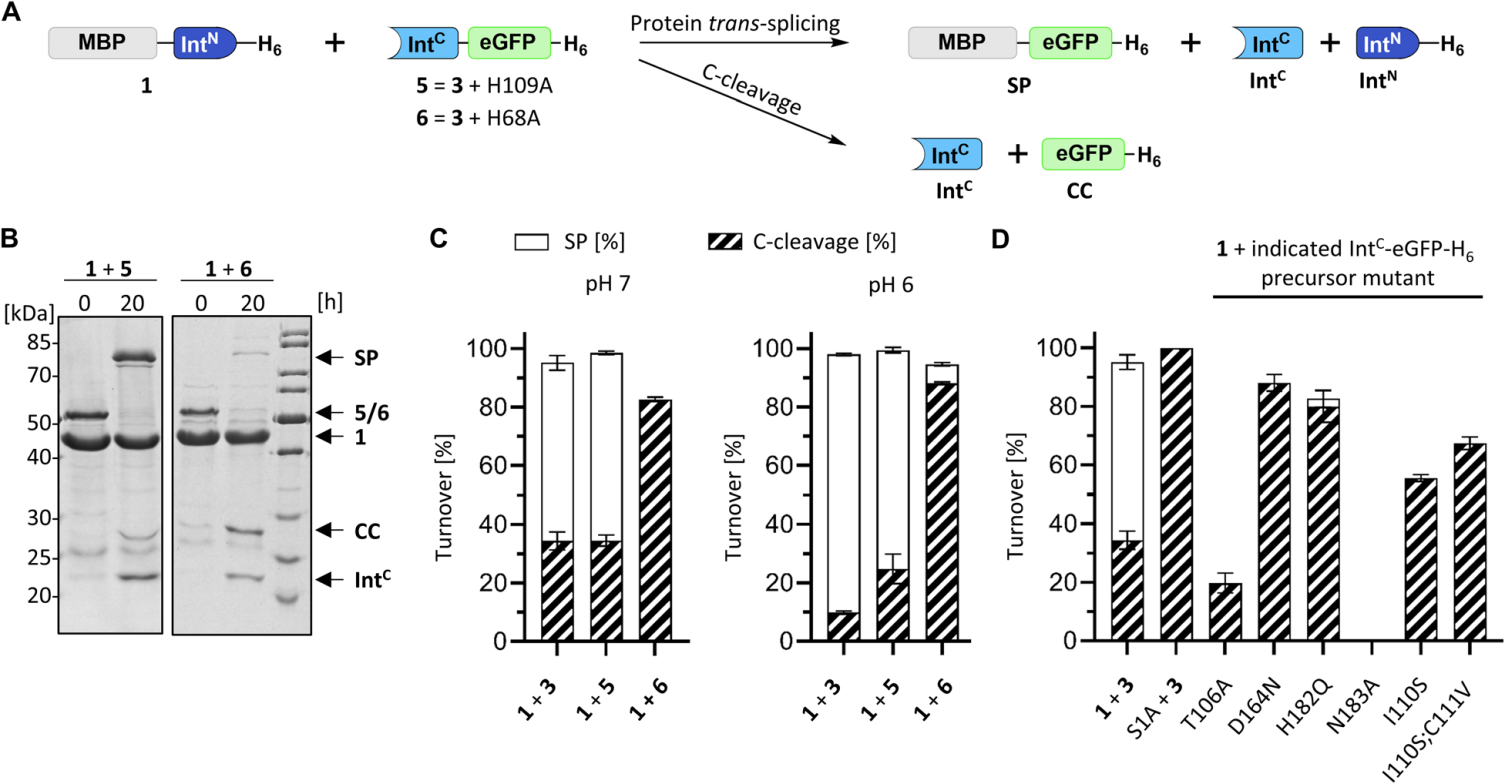
Mutational analysis of the PolB16 intein. (A) Scheme of the PTS reactions. Reaction were performed by mixing the Int^N^ (10 μM) and Int^C^ precursors (5 μM) at 25 °C at the indicated pH. (B) Representative SDS-PAGE analysis of reactions of the indicated mutants at pH 6. Shown are coomassie-stained gels. (C and D) Diagram representation of the reaction yields of the total turnover as the sum of the SP (white) and CC (diagonally striped) (*n* = 3; error bars represent standard deviations). See Fig. S5† for the SDS-PAGE analyses of the remainder of the mutants.

We next examined the origins of the observed C-terminal cleavage. At pH 7.0 the level of this side reaction is higher than typically observed for naturally split inteins. C-cleavage is caused by premature asparagine cyclization. Given the absence of any detectable N-cleavage as a potential initial trigger,^33^ we suspected that the asparagine cyclization step is partially decoupled from the initial N–O acyl shift within the protein splicing pathway.^34–36^ To probe the effect of a completely impaired initial N–O acyl shift we mutated the motif N3:10 histidine (His109), which is typically involved in this step by weakening the N-terminal scissile bond.^37–39^ Surprisingly, no significant adverse impact was observed with the Int^C^(H109A)-eGFP-H_6_ mutant (construct 5), despite the highly conserved nature and usually critical role of this residue in other inteins. The obtained splicing and C-cleavage yields remained nearly unchanged relative to the unmutated cysteine-less Int^C^ precursor (Fig. 5A–D). At pH 7 the splicing rate of the mutant precursor even increased by nearly 3-fold (0.24 ± 0.01 × 10^−3^ s^−1^), whereas at pH 6 a nearly 4-fold decrease was observed compared to the unmutated precursor (0.11 ± 0.01 × 10^−3^ s^−1^). Beyond the surprising dispensability of the motif N3:10 histidine, these findings hinted at unusual molecular properties.

### PolB16 intein crystal structures show the hallmarks of a class 1 intein but the region around the motif N3 His109 is unresolved

To better understand the molecular details of the splicing mechanism with a dispensable motif N3:10 histidine, we determined crystal structures of the PolB16 intein. To this end, we genetically fused the Int^N^ and Int^C^ precursors, each with 10 extein residues, with a 3 amino acid linker (GSH). Ser1 and Asn183 at the splice junctions were mutated to alanine to block all splicing activity. We obtained protein crystals in this format for both the wild-type sequence and the cysteine-less double mutant (C111A, C165A) and could solve these at resolutions of 1.85 and 2.6 Å, respectively. Both structures were virtually identical (0.185 Å RMSD, root mean square deviation), consistent with the negligible functional impact of mutating the non-conserved cysteine residues (Fig. S6†). The structures show the canonical horseshoe fold of inteins with an insertion in the region aa T84-D101 that explains the longer Int^C^ precursor compared to the minimal intein fold and other inteins (Fig. S7†). No sufficient electron density was found for a part within this region, D86-S95, suggesting it is mobile or less structured, as well as for the termini represented by extein residues (except Asp-1) and the ultimate N183A.

**Fig. 6:**
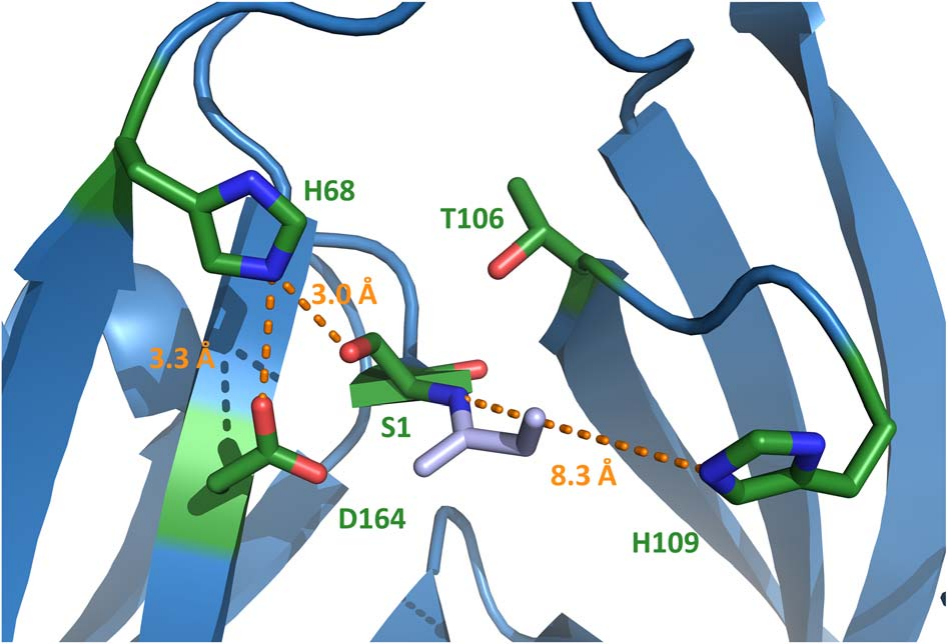
Crystal structure of the N-terminal active site of the PolB16 intein. The discussed and conserved residues of splicing motifs are highlighted in green. The intein was crystallized with S1A and N183A substitutions. For this representation, the side chain of Ser1 was modeled back into the structure using PyMol. Selected distances are given in Ångström (orange). Motif N3 histidine 109 is in unusual position.

Surprisingly, electron density was also missing for the stretch I110–N127 (H109–N127 for the cysteine-less variant), which involves the residues directly following the afore-mentioned catalytic motif N3:10 residue His109 and the next layer of amino acids typically wrapped around this part of the active site (Fig. S6†). The last well-resolved amino acids, Asp108 and His109, show a conformation of the polypeptide chain that departs from the canonical intein structure. As a consequence, His109 is placed in an unusual position, remote from the scissile bond (Fig. 6 and S7C†), which is incompatible with its usual catalytic role. This finding explains the dispensable role of the His109 residue for catalysis. Thr106 as the last residue found in the usual conformation (Fig. S7C†) is the highly conserved motif N3:7 residue that plays a critical role in most inteins by twisting the upstream scissile bond in a strained conformation to support the acyl shift reaction.^40^ Other key residues known to be involved in the canonical splicing mechanism were found at their usual locations, including the motif C2 Asp164, the motif C1 His182 as well as the mutated S1A at the splice junction (Fig. 6 and S7C†). Probing several conserved motif residues by site-directed mutagenesis (S1A, T106A, D164N, H182Q, and N183Q) revealed either entirely or almost completely impaired splicing activities, either at the expense of increased cleavage reactions or resulting in decreased intein turnover altogether (Fig. 5D and S5†). Together, these findings corroborated the canonical roles of the conserved residues. Only the unusual positioning and functional dispensability of the motif N3:10 residue His109 suggested a mechanistic deviation.

### Discovery of the novel motif NX with a histidine residue conserved only in cysteine-less inteins

Trying to explain the unusual functional and structural detachment of motif N3:10 residue His109 from the splicing pathway and the intein's active site, we noted in the crystal structures that His68 is positioned in the vicinity of the upstream splice junction. It approaches the N-terminal scissile bond from the opposite side as the motif N3:10 histidine (Fig. 6, S7C and D†). To our knowledge, a histidine at this position has not been probed by site-directed mutagenesis so far. We found that a H68A mutant of the Int^C^ precursor (construct 6) was indeed completely incapable of protein *trans*-splicing at pH 7.0, while only residual activity of about 5% was observed at pH 6.0 (Fig. 5B and C), indicating a critical role for this residue.

**Fig. 7:**
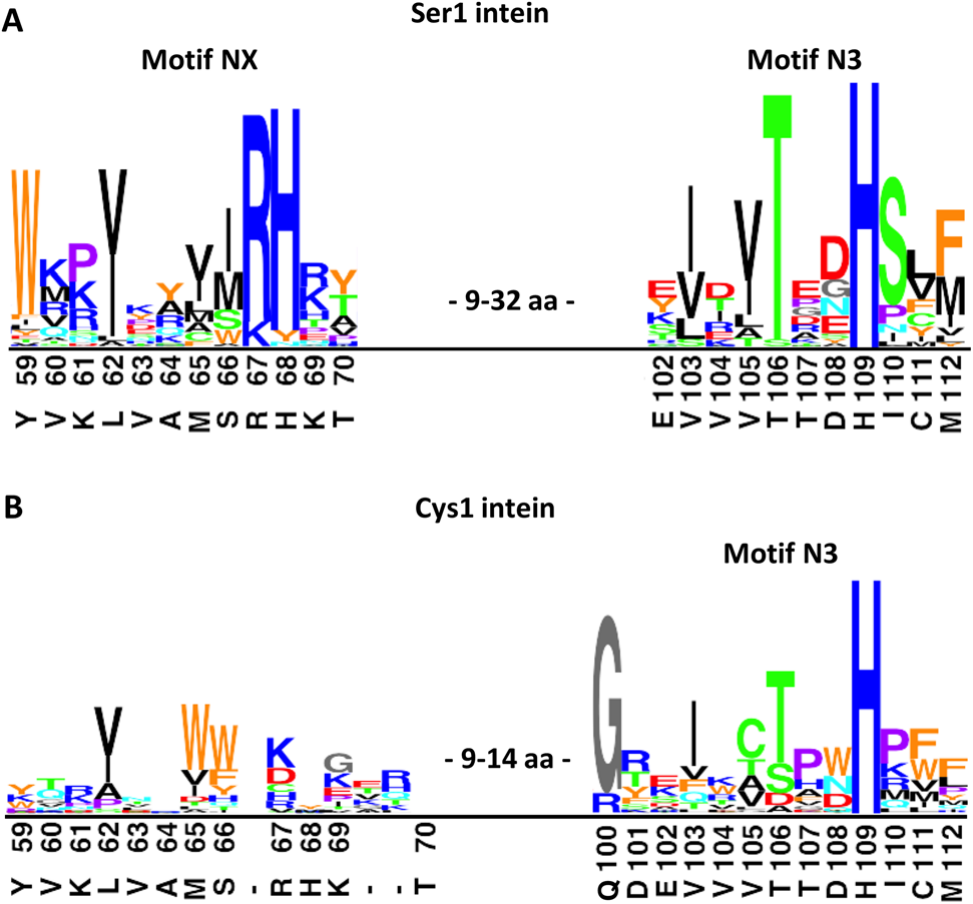
Sequence logo motifs N3 and NX in different intein types. The height of each individual amino acid represents their abundance at the given position. Amino acids were colored based on their properties. Corresponding positions and residues in PolB16 are shown below. (A) 58 Ser1 inteins from the InBase^43^ database that also have a Ser+1 or Thr+1 at the C-terminal splice junction. (B) 19 representative Cys1 inteins of known structure. PolB16 is part of the data for (A), but not for (B). Fig. S7† shows the multiple sequence alignments used to create these logos and the intervening regions between the NX and N3 regions.

To further examine the importance of His68, we constructed a multiple sequence alignment of the subgroup of known class 1 inteins that lack cysteines at the intein junction positions, *i.e.*, using only Ser1/Ser+1 and Ser1/Thr+1 inteins (Fig. 7A and S7A†). These 58 inteins are highly diverse in sequence (Fig S7A†) and are of 16 different intein alleles (*e.g.*, integration points^41^). Strikingly, this analysis revealed that the histidine corresponding to His68 in the PolB16 intein is highly conserved in this cysteine-independent subset of inteins. In contrast, when aligning sequences of 19 representative inteins of known structure of the Cys1 subset (containing Cys+1, Ser+1 or Thr+1 at the C-terminal splice junction), including 15 different alleles, we found no histidine or another residue at this position to be conserved (Fig. 7B, S7B and C†), despite the fact that their backbone folds of the region around the histidine are very similar to the PolB16 intein. Thus, it appears that the newly identified histidine residue was so far overlooked, because in mixed multiple sequence alignments of Ser1 and Cys1 inteins, in which the former group represents the smaller subset of known sequences, it remained obscured. We term the new sequence motif with the unveiled conserved histidine in Ser1 intein the NX motif. It represents a new signature motif specific to class 1 inteins with oxyester chemistry (Fig. 7).

The NX motif histidine residue is invariably preceded by an arginine or lysine residue, which interacts with a conserved glutamic acid (Glu43 in the PolB16 intein) in a preceding β-strand (Fig. S7A and D†). Two of the examined sequences have tyrosine or asparagine residues instead of histidine, indicating that even in the Ser1 subset the motif NX histidine is not completely conserved, like all important intein residues except for those directly involved in the (thio)ester intermediates.

We next examined the role of motif NX histidine in two other Ser1 inteins (Fig. S8†). Its mutation to alanine in the previously reported Psp Pol-1 maxi-intein^17,42^ as well as Mvu-M7 Pol-3 mini-intein, taken from Inbase^43^ and reported here for the first time, virtually completely impaired splicing, suggesting a critical role. Of note, in both these inteins also the motif N3 histidine was critical, in contrast to the PolB16 intein. This latter histidine is absolutely conserved in all the Ser1 inteins we examined (Fig. 7 and S7†). Together, these findings underline the general importance of the newly discovered motif NX histidine contribution to catalysis in Ser1 inteins.

### Structure-guided engineering improves protein *trans*-splicing properties of the PolB16 intein

With the crystal structure as a basis, we attempted to rationally improve the splicing properties of the PolB16 intein. We focused on the surprisingly unstructured region around the N3 motif. Positions Ile110 and Cys111, following the conserved His109, are typically occupied in class 1 inteins by proline or a polar amino acid and a large hydrophobic or aromatic amino acid, respectively (Fig. 7).^29^ However, attempts to redesign this sequence stretch with I110S and I110S/C111V mutations, inspired by the corresponding residues of the Aes123 intein,^12^ led to inactive variants that only showed increased C-cleavage activity (∼0–90%; Fig. 5D and S5†).

**Fig. 8:**
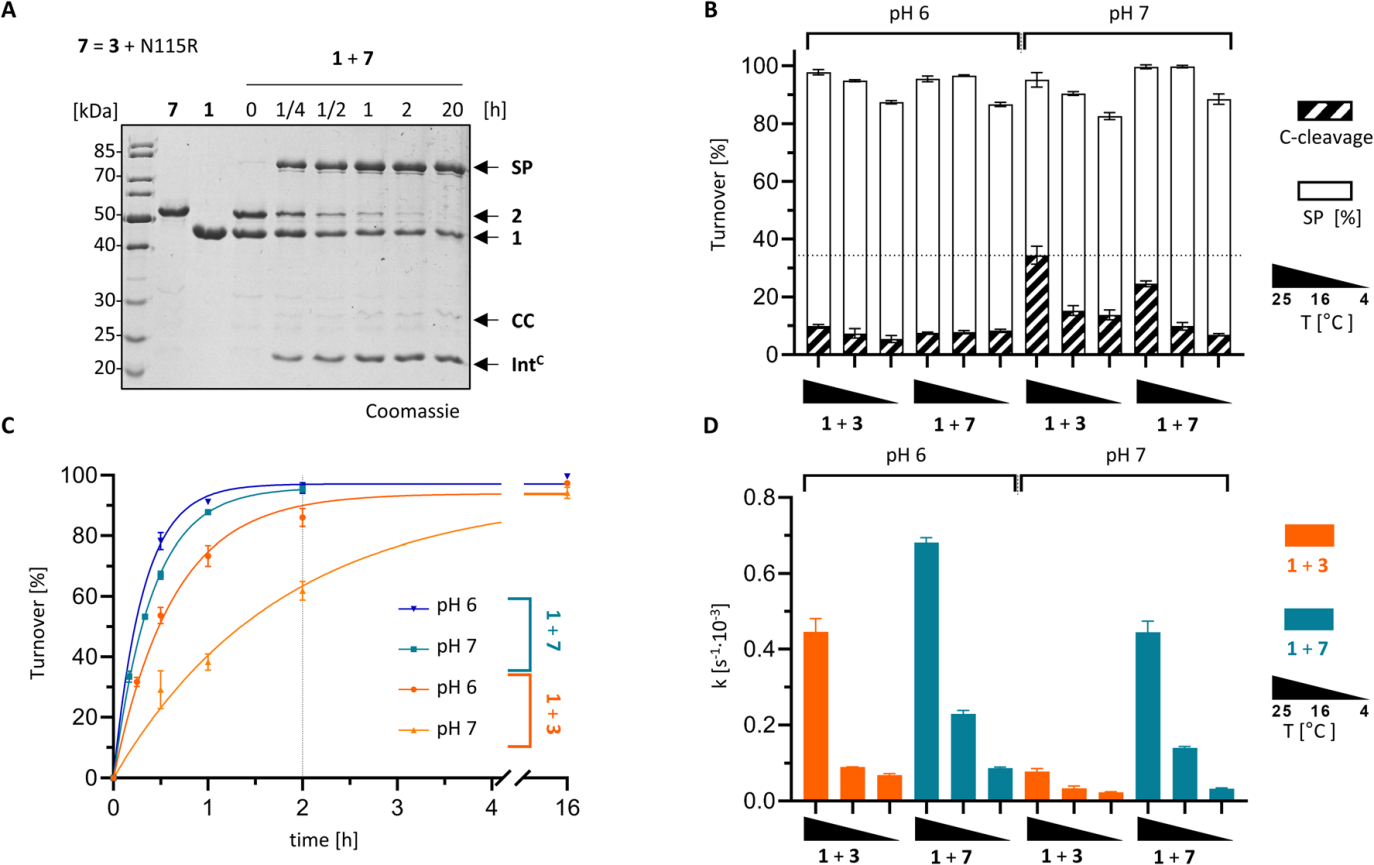
Rationally improved PolB16 intein with N115R mutation. PTS reactions of the Int^C^ precursors CL-Int^C^(C111A, N115R, C165A, C+4A)-eGFP-H_6_ (7) and Int^C^-eGFP-H_6_ (3) were performed according to the scheme shown in Fig. 3A using MBP-Int^N^-H_6_ (1) at 10 μM and the Int^C^ precursors at 5 μM. (A) Representative coomassie-stained SDS-PAGE gels of the reaction at 25 °C and pH 6. (B) Diagram of the total turnover as the sum of the SP (white) and CC (diagonally striped). Integrated into the same diagram is splicing rate (black; see *y*-axis on the right hand side) for the indicated split intein combinations (*n* = 3). (C) Time-courses of the reactions at 25 °C. (D) Kinetic rates of the turnover reactions as determined by densitometric gel analysis. Error bars in (B–D) represent standard deviations (*n* = 3).

We then analyzed a structural model of the PolB16 intein calculated using the AlphaFold algorithm.^44^ This model fitted our experimental data very well (Fig. S6†), interestingly however, it also predicted the typical intein fold for the region I110–N127 that was found unstructured in our crystals, albeit with only modest confidence scores (Fig. S9†). The lowest confidence was found in the turn between two β-sheets involving residues N115–H118. Guided by the multiple sequence alignment of Ser1/Ser(Thr+1) inteins (Fig. S7A†), we introduced an N115R substitution into the PolB16 intein. To our delight, this mutant Int^C^ precursor Int^C^(C111A, N115R, C165A)–eGFP-H_6_ (7) exhibited reduced C-terminal cleavage at pH 7.0 and significantly higher protein *trans*-splicing rates at both pH 7.0 and 6.0 (0.45 ± 0.02 × 10^−3^ s^−1^ and 0.68 ± 0.01 × 10^−3^ s^−1^; corresponding to *t*_½_ = 26 and 17 min, respectively; see Fig. 8). The rate at pH 7.0 and 25 °C represents a 6-fold improvement over the wild-type intein. This successful engineering result also further supported the idea that the region following motif N3 Thr106, including His109 and the unresolved stretch I110–N127, is attenuating the activity of the PolB16 intein.

## Conclusions

The novel atypically split PolB16 OarG intein reported herein is only the second reported mesophilic cysteine-less split intein. Although the native intein is not fully cysteine-less *per se*, its key catalytic residues contain no cysteine. Through its Ser1 and Ser+1 residues at the splice junctions, it forms oxyesters in the linear and branched intermediates of the protein splicing pathway. After replacing two non-conserved cysteines, we showed protein *trans*-splicing efficiency of ≥90% at pH 6.0 and, importantly, without having treated the intein precursors at any time with reducing agents. A significantly improved rate and slightly improved yields were achieved with the rationally engineered N115R mutant variant. These results also suggest that further improvement of the PolB16 intein will be possible. While the splicing efficiencies of the current engineered PolB16 intein mutant are useful for the preparative manipulation of purified proteins, its splicing rates are probably not ideal to trigger rapid biological processes occurring on the second-to-minute timescale in real time.^5,45–47^ For reactions at 37° the increasing tendency of the intein to form the C-terminal cleavage product at higher temperatures should be considered (Fig. S2†). Yet, protein *trans*-splicing also occurs at 37 °C (Fig. S2†) and a co-expression experiment of the PolB16 intein precursors in live *E. coli* cells indicated the compatibility of protein *trans*-splicing with a complex cellular environment (Fig. S10†), suggesting the intein will also be active in mammalian cells. Here we have focused on 25 °C as a useful temperature to modify purified proteins. The PolB16 intein is also fully orthogonal to the cysteine-less CL intein.^12^ Furthermore, the PolB16 intein is the first atypically split cysteine-less intein. Its Int^N^ fragment of only 15 aa is the shortest characterized so far^28^ and enables simple accessibility of synthetic and chemically functionalized Int^N^ precursors by SPPS for semi-synthetic protein splicing. Collectively, the PolB16 intein holds excellent potential for various protein engineering applications.

Our solved PolB16 intein crystal structures provide the first structural insight into a mesophilic cysteine-less split intein. A crystal structure of the thermophilic split Neq Pol intein was previously determined.^48^ Interestingly, in both these structures, the sequence region surrounding the motif N3:10 histidine was missing, suggesting an unfolded character. Surprisingly, our mutational analysis revealed this histidine to be dispensable for catalysis in the PolB16 intein. These findings seem to show a structural and functional correlation for the PolB16 intein. A similar argument cannot be made for the Neq Pol intein as no mutational studies have been reported for the latter, perhaps because of its poor behavior in recombinant protein production and purification.^16,48^ Typically, in other Ser1 inteins the motif N3 histidine is essential,^17^ as we have also shown here for the newly characterized Mvu-M7 Pol-3 mini-intein.

The remainder of the conserved active site residues in the PolB16 intein as well as our structural and biochemical analysis strongly suggest this intein follows the regular oxyester intermediates of a class 1 protein splicing pathway. Other *cis*-inteins with Ser1/Ser+1(Thr+1) residues have previously been functionally characterized^42^ and show similar active site arrangements, *i.e.*, the PI-TkoII intein^51^ and the MjaTFIIB intein, respectively.^52^

Surprisingly, despite this large body of knowledge on both Ser1 and Cys1 inteins, we newly identified an NX motif with a highly conserved histidine as the first signature sequence of the Ser1/Ser+1(Thr+1) subgroup of inteins. Its occurrence in inteins that utilize oxyester chemistry in their intermediates (and its absence in other inteins) has been overlooked so far. While in the PolB16 intein the motif NX histidine is critical and the motif N3 histidine is dispensable, the high conservation of both histidines in the Cys-independent Ser1 inteins suggests important roles for both these histidines in general (Fig. S7A and D†). This notion was further corroborated by the two other Ser1 inteins we have tested. Thus, the PolB16 intein seems to be a peculiar outlier with regard to the dispensable motif N3 histidine and represents another example for the tremendous diversity of variations of the protein splicing mechanism. The motif NX histidine appears to reflect the specialized environment evolved for the active site of inteins with a Ser1 residue.

Remarkably, the NX motif region has the same backbone fold as in highly diverse Cys1 inteins (Fig. S7C†), indicating that the NX histidine and other residues within the motif utilize the general intein fold to modify protein splicing catalysis for the special case of Ser1.

What is the contribution of the motif NX histidine to catalysis? Soon after the discovery of intein protein splicing^53,54^ in 1990 their catalytic mechanism was predicted to include an active site histidine by analogy to the catalytic triad mechanism of cysteine and serine proteases.^55,56^ The few known examples prompted the suggestion of the conserved penultimate histidine to be this residue. However, computational analysis of the then 10 known intein sequences showed a newly discovered conserved histidine of motif N3 (then termed block B) as a better candidate for the theoretical active site histidine.^31^ This was later verified by a genetic screen and intein structure determination.^57,58^ However, while some work points to the N3 histidine as a general base for the nucleophilic side chain from residue 1,^38,59^ there is also ample evidence for an alternative role in destabilizing and twisting the upstream scissile bond.^37,39,49,50^ This evidence includes the notion that the N3 histidine is typically facing the peptide backbone and not the position 1 side chain. Given the different nucleophilicities of the cysteine and serine side chains, a higher degree of activation would be required for a Ser1 residue. For example, Ser1 inteins could have evolved to rely on the assistance of a histidine as a general base histidine, while Cys1 inteins have not. Similarly, cysteine and serine hydrolases exhibit specialization with differently tuned catalytic dyads and triads, in which the attacking nucleophilic side chains are not simply interchangeable.^60^

Our PolB16 intein structures showed the motif NX histidine in possible hydrogen-bond forming distance to the Ser1, when the side chain hydroxyl of the latter is modeled back in (3.0 Å; Fig. 6). Similar observations can be made for the other known crystal structures of Ser1 inteins.^48,51,52^ Therefore, it is most tempting to postulate that the motif NX histidine assists in the initial N–O acyl shift by polarizing the Ser1 hydroxyl group to facilitate the attack on the backbone carbonyl carbon of the upstream scissile bond. A similar suggestion was made by Ribo and co-workers for the corresponding His61 of the thermophilic Neq Pol intein, however, without performing any site-directed mutagenesis to substantiate the hypothesis.^48^ Alternatively, these histidines might deprotonate or help position the conserved motif C2 aspartate (Asp164 in the PolB16 intein in a distance of 3.3 Å), which is implicated in the N–S (or N–O) acyl shift in other inteins. Furthermore, it might be involved in the transesterification step by helping in the attack of the Ser+1/Thr+1 nucleophile through further polarization of the Ser1 ester carbonyl group. The lack of the motif NX histidine in the GOS TerL intein,^20^ the only known Ser1 intein with a more reactive Cys+1 nucleophile that may be less dependent on such additional catalysis, would be consistent with this latter idea. Future work is required to study the exact catalytic role of the motif NX histidine.

In summary, the cysteine-less split PolB16 intein holds great potential for versatile splicing applications. Our identification of the motif NX histidine helps explain the specialized catalysis of Ser1/Ser+1(Thr+1) inteins. It might also enable new approaches to engineer powerful, new cysteine-less inteins. In general, we expect cysteine-less inteins to prove very useful in the future for applications to be performed in the absence of reducing agents or in combination with other thiol-based chemistry schemes that could not be carried out with Cys-dependent inteins or alternative cysteine-dependent protein ligation tools.^61,62^

## Data availability

ESI is available comprising additional experimental and sequence data. Coordinates of the crystal structures of the wildtype and C111A/C165A mutant of the PolB16 intein have deposited at the Protein Data Bank under pdb accessions numbers 8CPN and 8CPO, respectively. Further original data is available from the authors upon request.†

## Author contributions

Conceptualization: T. P., S. P., H. D. M.; formal analysis: T. P., A. S., S. K., D. K., H. D. M.; funding acquisition: H. D. M.; investigation: T. P., A. S., S. K., M. E., S. P., H. D. M.; project administration: S. P., D. K., H. D. M.; supervision: H. D. M.; writing: T. P., S. P., D. K. and H. D. M.

## Conflicts of interest

There are no conflicts to declare.

## Supplementary Material

SC-014-D3SC01200J-s001
