# Supplementary material for: Structural and biochemical analysis of a novel atypically split intein reveals a conserved histidine specific to cysteine-less inteins
Source: Chem Sci. 2023 Apr 24;14(19):5204–13. doi: 10.1039/d3sc01200j (PMC10189870; doi:10.1039/d3sc01200j)
Supplement: SC-014-D3SC01200J-s001 [file SC-014-D3SC01200J-s001.pdf]

Electronic Supplementary Material (ESI) for Chemical Science.
This journal is © The Royal Society of Chemistry 2023

Electronic Supplementary Information (ESI)

Structural and biochemical analysis of a novel atypically split intein reveals a conserved histidine specific to cysteine-less inteins

Tim Pasch,[1] Alexander Schröder,[1] Sabrina Kattelmann,[1] Miriam Eisenstein,[2] Shmuel Pietrokovski,*[2] Daniel Kümmel*[1] and Henning D. Mootz*[1]

[1]Institute of Biochemistry, University of Muenster, Corrensstr. 36, 48149 Münster, Germany

[2]Department of Molecular Genetics, Weizmann Institute of Science, Rehovot 76100, Israel

* = corresponding authors:

E-mail: Henning.Mootz@uni-muenster.de, Daniel.kuemmel@uni-muenster.de, Shmuel.pietrokovski@weizmann.ac.il

Table of Contents

METHODS 2
- General 2
- Protein production and purification 2
- Splice assays 3
- Densitometric analysis and determination of rate constants 3
- Solid phase peptide synthesis of Fluorescein-PB16^N 4
- Mass spectrometry 4

SUPPORTING TABLES 5
- Table S1 Data collection and refinement statistics for the PolB-16_OarG wild-type and the cysteine-free split intein 5
- Table S2: List of recombinantly produced proteins and synthesized peptide used in this study 6
- Table S3: Sequences of recombinantly produced proteins and synthesized peptides 7

Supporting Figures 11

Supporting References 20

METHODS

General

Solvents and standard chemical reagents were purchased from Sigma Aldrich, Acros Organics, TCI, Alfa Aesar, Carbolution, Fluorochem, Iris or Merck and were used without further purification. Restriction enzymes were purchased from Thermo Scientific. Synthetic DNA strings were ordered from Thermo Fisher. Synthetic oligonucleotides were ordered from Biolegio. Plasmids were verified by DNA sequencing by Seqlab.

Computational sequence analyses

PolB16 was identified by searching for intein motifs as previously described[1] in a dataset of sheep gut metagenomes (GenBank accession AUXO010000000). GenBank accession and coordinates for the Int^N and Int^C intein regions are AUXO013913591.1:1843-1887 and AUXO013913591.1:4443-4901+AUXO012578971.1:1-83, respectively.[2] The NX motif was generated using the glam2 program[3] on intein sequences from the InBase database[4] that did not have cysteines in both 1 and +1 positions, and were not class-3 inteins.[1] Corresponding regions to the NX motif in Cys1 inteins were identified by superposition of the PolB16 structure NX region on representative known structures of Cys1 class-1 inteins (excluding inteins with redundant or engineered sequences). PolB16 Cα atoms positions of residues 59-69, for the NX motif, and 101-106, for the N3 motif, and corresponding positions of other structures (e.g., 7OEC 61-71 and 85-96, where the later segment is extended by 6 residues in all structures) were used in the superposition. Once superposed, the position of each residue in the intervening segment was compared to all other structures and a residue was considered aligned if its Cα atom was within 1.5Å of the Cα of a residue in another protein and their Cα-Cβ vectors pointed in the same direction. Sequence logos of protein multiple sequence alignments were created as previously described.[1]

Protein production and purification

All proteins were produced in *E. coli* BL21(DE3) Gold cells. Cells were cultured at 37 °C in LB-medium with the corresponding antibiotic until an OD_{600} of 0.6 – 0.8 was reached. Protein expression was induced at 20 °C for 20 h by either adding IPTG (0.4 mM, pET-based vector systems) or L-Arabinose (0.2 % w/v, pBAD-based vector systems). Cell pellets were collected by centrifugation, resuspended in the respective purification buffer, flash frozen and stored at -20 °C till further use. Resuspended cells were ruptured using an Emulsiflex C5 emulsifier (Avestin). Insoluble fractions were removed by centrifugation and the supernatant fractions were used to purify the proteins.

For purification via Ni-NTA affinity chromatography of His-tagged proteins, cell pellets were resuspended in Ni-NTA buffer (50 mM Tris/HCl, 300 mM NaCl, pH 8.0). Purification was performed at 4 °C using flow gravity flow columns with a bed volume of 1 mL of Ni-NTA resin (Cube Biotech). For washing, two steps with Ni-NTA buffer with Ni-NTA buffer + 40 mM imidazole were performed. Proteins were eluted in a single fraction (2 mL) with Ni-NTA buffer + 250 mM imidazole.

For purification via size-exclusion chromatography (SEC) the protein solution was injected onto a HiLoad 16/600 Superdex 200 prep grade column at 4 °C using an ÄKTA Purifier (GE Healthcare). The proteins were eluted at a flow rate of 1 mL/min. Fractions were collected and

upconcentrated. Purified proteins were dialyzed three times against a PBS buffer and finally dialyzed against PBS buffer + 10 % glycerol before flash freezing in liquid nitrogen and storage at -80 °C. Protein concentrations were determined using the calculated extinction coefficient at 280 nm.

Constructs for crystallization (**30** and **31**) were purified via chitin-binding domain (CBD) pulldown using the IMPACT kit (Intein mediated Purification with an Affinity Chitin-binding tag; *New England Biolabs*). The supernatant of the centrifuged cell lysate was transferred to a gravity flow column with chitin-agarose. This step was followed by a wash step with 10 CV CBD buffer (Tris/HCl 20 mM, NaCl 500 mM, EDTA 1 mM, pH 8.0). The N-terminal cleavage of the fused Ssp $GyrB^N$ intein[5] and the subsequent release of the protein of interest was induced by the addition of 5 CV cleavage buffer (CBD-Buffer + 100 mM DTT). The column with the cleavage buffer was left at 4 °C shaking for 48 hours. Afterwards the eluate was collected, and the column was again eluted with 5 CV cleavage buffer. The two elution fractions were united and concentrated for further use.

For purification of Psp GBD-Pol intein precursor constructs affinity chromatography on an amylose resin (NEB) was performed. 2 g/L glucose was added to the LB medium (300 mL) before and after induction of protein expression to prevent the expression of amylase. Protein expression was induced at 20 °C for 20 h by adding IPTG 0.4 mM. Cell pellets were resuspended in ACB buffer (20 mM Tris, 200 mM NaCl, 1 mM EDTA, 1 mM DTT, pH 7.4) and lysed using an Emulsiflex C5 emulsifier (Avestin). Purification was performed at 4 °C using gravity flow columns with a bed volume of 1.5 mL resin. The column was washed with 10 column volumes of ACB buffer. The protein was eluted in three fractions containing 1 mL of column buffer + 10 mM maltose.

Recombinant precursor protein expression of the Mvu-M7-Pol-3 intein and its histidine mutants was induced at 28 °C for 10 h by adding IPTG (0.4 mM). Proteins were then purified by Ni-NTA affinity chromatography as described above.

Splice assays

Protein trans-splicing assays were performed in PBS using the described concentrations and at the mentioned temperatures. For determination of splicing rates, one of the split intein precursors was used at either a three- or four-fold excess in order to carry out the splicing reaction under pseudo-first order conditions. The splicing reaction was initiated by mixing N- and C-terminal intein precursors. The reaction was stopped at the described time points by taking an aliquot of the reaction mixture and boiling (5 min, 98 °C) the aliquots in 4x SDS sample buffer (500 mM Tris/HCl, 8 % (w/v) SDS, 40 % (v/v) glycerol, 20 % (v/v) 2-mercaptoethanol, 5 mg mL^{-1} bromophenol blue, pH 6.8).

Cis-splicing assays were conducted *in vivo* in *E. coli* cells. The *cis*-constructs were expressed at 20 °C for 20 h and the splice product was purified according to the purification methods in the preceding paragraph.

Densitometric analysis and determination of rate constants

Coomassie-stained SDS gels were scanned and the signal intensity of Coomassie-stained bands was determined using ImageJ. The signal intensity was normalized to the molecular weight of the protein. The normalized intensities of the splice product (SP), C-Cleavage (CC) and precursor protein (Int^C) were calculated and inserted in the following equations to determine the desired values, including the absolute turnover:

$$SP[\%] = \frac{100}{1+\frac{Int^C+CC}{SP}} \quad CC[\%] = \frac{100}{1+\frac{Int^C+SP}{CC}} \quad Turnover[\%] = SP[\%] + CC[\%]$$

The splice yield was plotted against the time and fitted to the following pseudo-first-order equation using GraphPad Prism (version 9.5):

$$P_t = P_0 * (1 - e^{-kt})$$

with P_t = yield of product at time t, P_0 = maximum yield of product, t = time and k = pseudo-first-order reaction constant.

Solid phase peptide synthesis of Fluorescein-Int^N (*CF*-Int^N)

The peptide was assembled on a TGR resin with a freshly coupled rink amide linker, by stepwise microwave assisted Fmoc-SPPS on a Liberty blue peptide synthesizer, operating on a 0.1 mmol scale. Activation of entering Fmoc-protected amino acids (Carbolution, Merck Millipore or Iris Biotech) was performed using Oxyma and DIC in DMF (1:1 molar ratio), with a 4 equivalent excess over the initial resin loading. Coupling steps were performed for initial 15 seconds at 75°C and 150 watts followed by 110 seconds at 90 °C and 30 watts. Fmoc-deprotection steps were performed by treatment of the resin with a 20% piperidine solution in DMF for initial 15 seconds at 75°C and 150 watts followed by 50 seconds at 90 °C and 30 watts. Following each deprotection step, the resin was washed thoroughly with DMF. 5(6)-Carboxyfluorescein (*CF*) was manually coupled to the peptide by adding a solution of 5(6)-carboxyfluorescein-OH (2 eq.) (Sigma Aldrich), DIC (2 eq.) and HOAt (2 eq.) in DMF to the resin and shaking at room temperature for 16 hours. The resin was subsequently washed with DMF and DCM, and dried under nitrogen flow. The labelled peptide was finally cleaved off the resin by treatment with an ice-cold TFA, TIS, water mixture (90:5:5) and allowed to shake at room temperature for 3 hours, followed by purification by RP-HPLC.

Mass spectrometry

The peptide CF-Int^N was analyzed using an Agilent 1260 Infinity series system (Agilent Technologies, Waldbronn, Germany) with a C18 column (ZORBAX SB-C18 RR HT, 3 x 50 mm, 1.8 µm, Agilent Technologies, Waldbronn, Germany).

Structure determination

For structure determination, two fusion constructs of the Int^N and Int^C fragments were used, either with or without the non-conserved cysteines mutated to alanine, each with 10 extein residues, connected by a GSH (Gly-Ser-His) linker and with Ser1 and Asn183 at the splice junctions mutated to Ala. Sitting drop crystallization was performed at 20 °C. The wildtype PolB16 variant with the non-conserved cysteines was used at 140 µM protein concentration. Best crystals grew in 0.1 M phosphate/citrate buffer pH 4.2, 38% ethanol, and 5% PEG1000. Crystals were soaked consecutively in reservoir solution plus 0.1 M and 0.2 M NaI for 2 h each, then transferred to cryo conditions with 60% ethanol and flash-frozen in liquid nitrogen. Diffraction data was collected at Helmholtz-Zentrum Berlin BL 14.2 (Ref[6]) and was processed with XDSAPP.[7] Initial phases were obtained by SAD (single wavelength anomalous diffraction, Phenix AutoSol)[8] and the model was generated by automated model building (Phenix AutoBuild),[9] followed by several rounds of manual building (coot)[10] and refinement (Phenix Refine).[11] The Cys-less version with the additional mutations C111A, C165A crystallized at 1.3 mM in the same conditions, but was transferred into mother liquor with 0.125 % (v/v) glutaraldehyde prior to vitrification in reservoir solution supplemented with 30% PEG 400. Diffraction data was collected at Helmholtz-Zentrum Berlin BL 14.1, processed with XDSAPP, and an

initial model obtained by MR (molecular replacement) with the wild-type structure (Phenix Phaser)[12] was finalized by several rounds of manual building (coot) and refinement (Phenix Refine).

Data collection and refinement statistics are summarized in Table SX, and the structure factors and models have been deposited to the PDB with accession numbers 8CPN (wild-type) and 8CPO (Cys-less).

SUPPORTING TABLES

Table S1 Data collection and refinement statistics for the PolB-16_OarG wild-type and the cysteine-free split intein

		**WT NaI soaked**		**Cysteine-less (d_2Cys)**	
Space group		$P3_2$ 2 1		$P3_2$ 2 1	
Wavelength [Å]		1.549800		0.976252	
Unit cell	a [Å]	69.42		68.91	
	b [Å]	69.42		68.91	
	c [Å]	79.16		79.1	
	α [°]	90.0		90	
	β [°]	90.0		90	
	γ [°]	120.0		120	
Resolution [Å] *		50 - 1.85	(1.96 -1.85)	50.0 -2.6	(2.75 – 2.6)
Reflections		36175	(5587)	7009	1109
Multiplicity *		9.3	(6.5)	19.2	18.5
I/σ *		19.31	(1.19)	37.59	3.3
Completeness [%] *		98.9%	(94.4)	99.6	98.6
R_{meas} [%] *		5.4	(122.7)	4.7	97.5
CC(1/2)		99.9	(72.1)	100	96.0
Wilson B factor [Å²]		40.65		86.78	
Refinement [Å]		33.1 - 1.85		47.6 – 2.6	
Reflections		36161		6998	
R_{work} / R_{free} [%]		21.1 / 24.7		24.4 / 28.27	
rmsd bond distances [Å]		0.016		0.011	
rmsd bond angles [°]		1.55		1.8	
Ramachandran diagram [%]					
favored		96.73		97.35	
allowed		3.27		2.65	
outlier		0.0		0.0	
B value [Å²]					
protein		61.65		109.37	
ligand		82.29		-	
waters		54.63		102.6	

Values in parenthesis refer to outer shell of reflections.

Table S2: List of recombinantly produced proteins and synthesized peptide used in this study

Protein/ peptide number	Name of construct	Encoding plasmid	Vector backbone	Reference
**1**	MBP-Int^{N}-H_6	pTP021	pMal-C2x	This work
**2**	Int^{C}-eGFP-H_6	pTP048	pBAD	This work
**3**	Int^{C}[C111A;C165A;C+4A]-eGFP-H_6	pTP223	pBAD	This work
**4**	*CF*-Int^{N} (synthetic peptide)	-	-	This work
**5**	Int^{C}[H109A;C111A;C165A;C+4A]-eGFP-H_6	pTP321	pBAD	This work
**6**	Int^{C}[H68A;C111A;C165A;C+4A]-eGFP-H_6	pTP352	pBAD	This work
**7**	Int^{C}[C111A;N115R;C165A;C+4A]-eGFP-H_6	pTP317	pBAD	This work
**8**	Int^{C}[C111A]-eGFP-H_6	pTP244	pBAD	This work
**9**	Int^{C}[C165A]-eGFP-H_6	pTP243	pBAD	This work
**10**	Int^{C}[C111A;C165A]-eGFP-H_6	pTP071	pBAD	This work
**11**	*CF*-Int^{N}(CL-Intein) (synthetic peptide)	-	-	Ref[13]
**12**	SBP-Int^{C}(CL-Intein)-eGFP-H_6	pTP096	pET16b	This work
**13**	MBP-Int^{N}[S1A]-H_6	pTP046	pMal-C2x	This work
**14**	Int^{C}[C111A;C165A;C+4A]-Trx-H_6	pTP061	pBAD	This work
**15**	Int^{C}[T106A;C111A;C165A;C+4A]-eGFP-H_6	pTP364	pBAD	This work
**16**	Int^{C}[I110S;C111A;C165A;C+4A]-eGFP-H_6	pTP315	pBAD	This work
**17**	Int^{C}[C111A;D164N;C165A;C+4A]-eGFP-H_6	pTP307	pBAD	This work
**18**	Int^{C}[C111A;C165A;H182Q;C+4A]-eGFP-H_6	pTP309	pBAD	This work
**19**	Int^{C}[C111A;C165A;N183Q;C+4A]-eGFP-H_6	pTP361	pBAD	This work
**20**	Int^{C}[I110S;C111V;C165A;C+4A]-eGFP-H_6	pTP316	pBAD	This work
**21**	SBP-Int^{C}(CL-Intein)[H68A]-eGFP-H_6	pTP371	pET16b	This work
**22**	SBP-Int^{C}(CL-Intein)[H90A]-eGFP-H_6	pTP372	pET16b	This work
**23**	SBP-Int^{C}(CL-Intein)[H68A;H90A]-eGFP-H_6	pTP388	pET16b	This work
**24**	MBP-Psp-Pol-1-Paramyosin	pSB067	pMIP	Ref[14]
**25**	MBP-Psp-Pol-1[H96A]-Paramyosin	pAS077	pMIP	This work
**26**	MBP-Psp-Pol-1[H73A]-Paramyosin	pAS085	pMIP	This work
**27**	MBP-Mvu-M7-Pol-3-Trx-H_6	pAS088	pMAL1MPI	This work
**28**	MBP-Mvu-M7-Pol-3[H64A]-Trx-H_6	pAS091	pMAL1MPI	This work
**29**	MBP-Mvu-M7-Pol-3[H86A]-Trx-H_6	pAS092	pMAL1MPI	This work
**30**	Ex^{N}-Int^{N}[S1A]-GSH-Int^{C}[N183A]-Ex^{C}-SspGyrB^{N}(1-150)-CBD	pTP105	pBAD	This work
**31**	Ex^{N}-Int^{N}[S1A]-GSH-Int^{C}[C111A,C165A,N183A]-Ex^{C}-SspGyrB^{N}(1-150)-CBD	pTP221	pBAD	This work
**32**	MBP-Int^{N}-H_6	pTP022	pMal-C2x	This work
**33**	Int^{C}-Trx	pTP022	pMal-C2x	This work

CF = 5(6)-Carboxyfluorescein

Table S3: Sequences of recombinantly produced proteins and synthesized peptides

Protein/peptide number	Amino acid sequence
**1**	MKTEEGKLVIWINGDKGYNGLAEVGKKFEKDTGIKVTVEHPDKLEEKFPQVAATGDGPDIIFWAHDRFGGYAQSGLLAEITPD<br>KAFQDKLYPFTWDAVRYNGKLIAYPIAVEALSLIYNKDLLPNPPKTWEEIPALDKELKAKGKSALMFNLQEPYFTWPLIAADGGY<br>AFKYENGKYDIKDVGVDNAGAKAGLTFLVDLIKNKHMNADTDYSIAEAAFNKGETAMTINGPWAWSNIDTSKVNYGVTVLP<br>TFKGQPSKPFVGVLSAGINAASPNKELAKEFLENYLLTDEGLEAVNKDKPLGAVALKSYEEELAKDPRIAATMENAQKGEIMPNI<br>PQMSAFWYAVRTAVINAASGRQTVDEALKDAQTNSSSNNNNNNNNNNLGIEGRISEFSGDTDSVHGKTHVFIRSIKNGSHH<br>HHHH
**2**	MQEAKIDIKSLYDSLAKKYDVQHKNSYEVIYPKGYEIKVLGNKYVKLVAMSRHKTQKHLVKIVVKSEKTIDSLDPIRQKSLLKKQD<br>EVVVTTDHICMVYNDDHFFENVNAKNLKVGNYVSVYDEASDKEVIGEIASIEDLGMTDDYVYDCEVDDDSHAFYASNILVHNS<br>QFCNGTVSKGEELFTGVVPILVELDGDVNGHKFSVSGEGEGDATYGKLTLKFICTTGKLPVPWPTLVTTLTYGVQCFSRYPDHM<br>KQHDFFKSAMPEGYVQERTIFFKDDGNYKTRAEVKFEGDTLVNRIELKGIDFKEDGNILGHKLEYNYNSHNVYIMADKQKNGIK<br>VNFKIRHNIEDGSVQLADHYQQNTPIGDGPVLLPDNHYLSTQSALSKDPNEKRDHMVLLEFVTAAGITLGMDELYKGSRSHHH<br>HHH
**3**	MQEAKIDIKSLYDSLAKKYDVQHKNSYEVIYPKGYEIKVLGNKYVKLVAMSRHKTQKHLVKIVVKSEKTIDSLDPIRQKSLLKKQD<br>EVVVTTDHI**A**MVYNDDHFFENVNAKNLKVGNYVSVYDEASDKEVIGEIASIEDLGMTDDYVYD**A**EVDDDSHAFYASNILVHN<br>SQF**A**NGTVSKGEELFTGVVPILVELDGDVNGHKFSVSGEGEGDATYGKLTLKFICTTGKLPVPWPTLVTTLTYGVQCFSRYPDH<br>MKQHDFFKSAMPEGYVQERTIFFKDDGNYKTRAEVKFEGDTLVNRIELKGIDFKEDGNILGHKLEYNYNSHNVYIMADKQKN<br>GIKVNFKIRHNIEDGSVQLADHYQQNTPIGDGPVLLPDNHYLSTQSALSKDPNEKRDHMVLLEFVTAAGITLGMDELYKGSRS<br>HHHHHH
**4**	*CF*-SGDTDSVHGKTHVFIRSIKN
**5**	MQEAKIDIKSLYDSLAKKYDVQHKNSYEVIYPKGYEIKVLGNKYVKLVAMSRHKTQKHLVKIVVKSEKTIDSLDPIRQKSLLKKQD<br>EVVVTTD**A**I**A**MVYNDDHFFENVNAKNLKVGNYVSVYDEASDKEVIGEIASIEDLGMTDDYVYD**A**EVDDDSHAFYASNILVHN<br>SQF**A**NGTVSKGEELFTGVVPILVELDGDVNGHKFSVSGEGEGDATYGKLTLKFICTTGKLPVPWPTLVTTLTYGVQCFSRYPDH<br>MKQHDFFKSAMPEGYVQERTIFFKDDGNYKTRAEVKFEGDTLVNRIELKGIDFKEDGNILGHKLEYNYNSHNVYIMADKQKN<br>GIKVNFKIRHNIEDGSVQLADHYQQNTPIGDGPVLLPDNHYLSTQSALSKDPNEKRDHMVLLEFVTAAGITLGMDELYKGSRS<br>HHHHHH
**6**	MQEAKIDIKSLYDSLAKKYDVQHKNSYEVIYPKGYEIKVLGNKYVKLVAMSR**A**KTQKHLVKIVVKSEKTIDSLDPIRQKSLLKKQD<br>EVVVTTDHI**A**MVYNDDHFFENVNAKNLKVGNYVSVYDEASDKEVIGEIASIEDLGMTDDYVYD**A**EVDDDSHAFYASNILVHN<br>SQF**A**NGTVSKGEELFTGVVPILVELDGDVNGHKFSVSGEGEGDATYGKLTLKFICTTGKLPVPWPTLVTTLTYGVQCFSRYPDH<br>MKQHDFFKSAMPEGYVQERTIFFKDDGNYKTRAEVKFEGDTLVNRIELKGIDFKEDGNILGHKLEYNYNSHNVYIMADKQKN<br>GIKVNFKIRHNIEDGSVQLADHYQQNTPIGDGPVLLPDNHYLSTQSALSKDPNEKRDHMVLLEFVTAAGITLGMDELYKGSRS<br>HHHHHH
**7**	MQEAKIDIKSLYDSLAKKYDVQHKNSYEVIYPKGYEIKVLGNKYVKLVAMSRHKTQKHLVKIVVKSEKTIDSLDPIRQKSLLKKQD<br>EVVVTTDHI**A**MVY**R**DDHFFENVNAKNLKVGNYVSVYDEASDKEVIGEIASIEDLGMTDDYVYD**A**EVDDDSHAFYASNILVHNS<br>QF**A**NGTVSKGEELFTGVVPILVELDGDVNGHKFSVSGEGEGDATYGKLTLKFICTTGKLPVPWPTLVTTLTYGVQCFSRYPDHM<br>KQHDFFKSAMPEGYVQERTIFFKDDGNYKTRAEVKFEGDTLVNRIELKGIDFKEDGNILGHKLEYNYNSHNVYIMADKQKNGIK<br>VNFKIRHNIEDGSVQLADHYQQNTPIGDGPVLLPDNHYLSTQSALSKDPNEKRDHMVLLEFVTAAGITLGMDELYKGSRSHHH<br>HHH
**8**	MQEAKIDIKSLYDSLAKKYDVQHKNSYEVIYPKGYEIKVLGNKYVKLVAMSRHKTQKHLVKIVVKSEKTIDSLDPIRQKSLLKKQD<br>EVVVTTDHI**A**MVYNDDHFFENVNAKNLKVGNYVSVYDEASDKEVIGEIASIEDLGMTDDYVYDCEVDDDSHAFYASNILVHNS<br>QFCNGTVSKGEELFTGVVPILVELDGDVNGHKFSVSGEGEGDATYGKLTLKFICTTGKLPVPWPTLVTTLTYGVQCFSRYPDHM<br>KQHDFFKSAMPEGYVQERTIFFKDDGNYKTRAEVKFEGDTLVNRIELKGIDFKEDGNILGHKLEYNYNSHNVYIMADKQKNGIK<br>VNFKIRHNIEDGSVQLADHYQQNTPIGDGPVLLPDNHYLSTQSALSKDPNEKRDHMVLLEFVTAAGITLGMDELYKGSRSHHH<br>HHH
**9**	MQEAKIDIKSLYDSLAKKYDVQHKNSYEVIYPKGYEIKVLGNKYVKLVAMSRHKTQKHLVKIVVKSEKTIDSLDPIRQKSLLKKQD<br>EVVVTTDHICMVYNDDHFFENVNAKNLKVGNYVSVYDEASDKEVIGEIASIEDLGMTDDYVYD**A**EVDDDSHAFYASNILVHNS<br>QFCNGTVSKGEELFTGVVPILVELDGDVNGHKFSVSGEGEGDATYGKLTLKFICTTGKLPVPWPTLVTTLTYGVQCFSRYPDHM<br>KQHDFFKSAMPEGYVQERTIFFKDDGNYKTRAEVKFEGDTLVNRIELKGIDFKEDGNILGHKLEYNYNSHNVYIMADKQKNGIK<br>VNFKIRHNIEDGSVQLADHYQQNTPIGDGPVLLPDNHYLSTQSALSKDPNEKRDHMVLLEFVTAAGITLGMDELYKGSRSHHH<br>HHH
**10**	MQEAKIDIKSLYDSLAKKYDVQHKNSYEVIYPKGYEIKVLGNKYVKLVAMSRHKTQKHLVKIVVKSEKTIDSLDPIRQKSLLKKQD<br>EVVVTTDHI**A**MVYNDDHFFENVNAKNLKVGNYVSVYDEASDKEVIGEIASIEDLGMTDDYVYD**A**EVDDDSHAFYASNILVHN<br>SQF**A**NGTVSKGEELFTGVVPILVELDGDVNGHKFSVSGEGEGDATYGKLTLKFICTTGKLPVPWPTLVTTLTYGVQCFSRYPDH<br>MKQHDFFKSAMPEGYVQERTIFFKDDGNYKTRAEVKFEGDTLVNRIELKGIDFKEDGNILGHKLEYNYNSHNVYIMADKQKN<br>GIKVNFKIRHNIEDGSVQLADHYQQNTPIGDGPVLLPDNHYLSTQSALSKDPNEKRDHMVLLEFVTAAGITLGMDELYKGSRS<br>HHHHHH

**11**	*CF*-YIDTDSVVGDTIIDVSGKKMTIAEFYDSTPD
**12**	MDEKTTGWRGGHVVEGLAGELEQLRARLEHHPQGQREPGASGGGGSSSEARDWVKRVGGKTSLSVNTYSGEVERKNINYI<br>MKHTVKKRMFKIKAGGKEVIVTADHSVMVKRDGKIIDVKPTEMKQTDRVVKWMLTGSHMIEFIEFEIEDLGVMEIDVYDIEV<br>DGNHNFFGNDILVHNSVYLNGTVSKGEELFTGVVPILVELDGDVNGHKFSVSGEGEGDATYGKLTLKFICTTGKLPVPWPTLVT<br>TLTYGVQCFSRYPDHMKQHDFFKSAMPEGYVQERTIFFKDDGNYKTRAEVKFEGDTLVNRIELKGIDFKEDGNILGHKLEYNYN<br>SHNVYIMADKQKNGIKVNFKIRHNIEDGSVQLADHYQQNTPIGDGPVLLPDNHYLSTQSALSKDPNEKRDHMVLLEFVTAAGI<br>TLGMDELYKGSRSHHHHHH
**13**	MKTEEGKLVIWINGDKGYNGLAEVGKKFEKDTGIKVTVEHPDKLEEKFPQVAATGDGPDIIFWAHDRFGGYAQSGLLAEITPD<br>KAFQDKLYPFTWDAVRYNGKLIAYPIAVEALSLIYNKDLLPNPPKTWEEIPALDKELKAKGKSALMFNLQEPYFTWPLIAADGGY<br>AFKYENGKYDIKDVGVDNAGAKAGLTFLVDLIKNKHMNADTDYSIAEAAFNKGETAMTINGPWAWSNIDTSKVNYGVTVLP<br>TFKGQPSKPFVGVLSAGINAASPNKELAKEFLENYLLTDEGLEAVNKDKPLGAVALKSYEEELAKDPRIAATMENAQKGEIMPNI<br>PQMSAFWYAVRTAVINAASGRQTVDEALKDAQTNSSSNNNNNNNNNNLGIEGRISEFSGDTD**A**VHGKTHVFIRSIKNGSHH<br>HHHH
**14**	MQEAKIDIKSLYDSLAKKYDVQHKNSYEVIYPKGYEIKVLGNKYVKLVAMSRHKTQKHLVKIVVKSEKTIDSLDPIRQKSLLKKQD<br>EVVVTTDHI**A**MVYNDDHFFENVNAKNLKVGNYVSVYDEASDKEVIGEIASIEDLGMTDDYVYD**A**EVDDDSHAFYASNILVHN<br>SQF**A**NGTGSDKIIHLTDDSFDTDVLKADGAILVDFWAHWCGPCKMIAPILDEIADEYQGKLTVAKLNIDHNPGTAPKYGIRGIP<br>TLLLFKNGEVAATKVGALSKGQLKEFLDANLAGSEFRSHHHHHH
**15**	MQEAKIDIKSLYDSLAKKYDVQHKNSYEVIYPKGYEIKVLGNKYVKLVAMSRHKTQKHLVKIVVKSEKTIDSLDPIRQKSLLKKQD<br>EVVV**A**TDHI**A**MVYNDDHFFENVNAKNLKVGNYVSVYDEASDKEVIGEIASIEDLGMTDDYVYD**A**EVDDDSHAFYASNILVHN<br>SQF**A**NGTVSKGEELFTGVVPILVELDGDVNGHKFSVSGEGEGDATYGKLTLKFICTTGKLPVPWPTLVTTLTYGVQCFSRYPDH<br>MKQHDFFKSAMPEGYVQERTIFFKDDGNYKTRAEVKFEGDTLVNRIELKGIDFKEDGNILGHKLEYNYNSHNVYIMADKQKN<br>GIKVNFKIRHNIEDGSVQLADHYQQNTPIGDGPVLLPDNHYLSTQSALSKDPNEKRDHMVLLEFVTAAGITLGMDELYKGSRS<br>HHHHHH
**16**	MQEAKIDIKSLYDSLAKKYDVQHKNSYEVIYPKGYEIKVLGNKYVKLVAMSRHKTQKHLVKIVVKSEKTIDSLDPIRQKSLLKKQD<br>EVVVTTDH**SA**MVYNDDHFFENVNAKNLKVGNYVSVYDEASDKEVIGEIASIEDLGMTDDYVYD**A**EVDDDSHAFYASNILVHN<br>SQF**A**NGTVSKGEELFTGVVPILVELDGDVNGHKFSVSGEGEGDATYGKLTLKFICTTGKLPVPWPTLVTTLTYGVQCFSRYPDH<br>MKQHDFFKSAMPEGYVQERTIFFKDDGNYKTRAEVKFEGDTLVNRIELKGIDFKEDGNILGHKLEYNYNSHNVYIMADKQKN<br>GIKVNFKIRHNIEDGSVQLADHYQQNTPIGDGPVLLPDNHYLSTQSALSKDPNEKRDHMVLLEFVTAAGITLGMDELYKGSRS<br>HHHHHH
**17**	MQEAKIDIKSLYDSLAKKYDVQHKNSYEVIYPKGYEIKVLGNKYVKLVAMSRHKTQKHLVKIVVKSEKTIDSLDPIRQKSLLKKQD<br>EVVVTTDHI**A**MVYNDDHFFENVNAKNLKVGNYVSVYDEASDKEVIGEIASIEDLGMTDDYVY**NA**EVDDDSHAFYASNILVHN<br>SQF**A**NGTVSKGEELFTGVVPILVELDGDVNGHKFSVSGEGEGDATYGKLTLKFICTTGKLPVPWPTLVTTLTYGVQCFSRYPDH<br>MKQHDFFKSAMPEGYVQERTIFFKDDGNYKTRAEVKFEGDTLVNRIELKGIDFKEDGNILGHKLEYNYNSHNVYIMADKQKN<br>GIKVNFKIRHNIEDGSVQLADHYQQNTPIGDGPVLLPDNHYLSTQSALSKDPNEKRDHMVLLEFVTAAGITLGMDELYKGSRS<br>HHHHHH
**18**	MQEAKIDIKSLYDSLAKKYDVQHKNSYEVIYPKGYEIKVLGNKYVKLVAMSRHKTQKHLVKIVVKSEKTIDSLDPIRQKSLLKKQD<br>EVVVTTDHI**A**MVYNDDHFFENVNAKNLKVGNYVSVYDEASDKEVIGEIASIEDLGMTDDYVYD**A**EVDDDSHAFYASNILV**Q**N<br>SQF**A**NGTVSKGEELFTGVVPILVELDGDVNGHKFSVSGEGEGDATYGKLTLKFICTTGKLPVPWPTLVTTLTYGVQCFSRYPDH<br>MKQHDFFKSAMPEGYVQERTIFFKDDGNYKTRAEVKFEGDTLVNRIELKGIDFKEDGNILGHKLEYNYNSHNVYIMADKQKN<br>GIKVNFKIRHNIEDGSVQLADHYQQNTPIGDGPVLLPDNHYLSTQSALSKDPNEKRDHMVLLEFVTAAGITLGMDELYKGSRS<br>HHHHHH
**19**	MQEAKIDIKSLYDSLAKKYDVQHKNSYEVIYPKGYEIKVLGNKYVKLVAMSRHKTQKHLVKIVVKSEKTIDSLDPIRQKSLLKKQD<br>EVVVTTDHI**A**MVYNDDHFFENVNAKNLKVGNYVSVYDEASDKEVIGEIASIEDLGMTDDYVYD**A**EVDDDSHAFYASNILVH**Q**<br>SQF**A**NGTVSKGEELFTGVVPILVELDGDVNGHKFSVSGEGEGDATYGKLTLKFICTTGKLPVPWPTLVTTLTYGVQCFSRYPDH<br>MKQHDFFKSAMPEGYVQERTIFFKDDGNYKTRAEVKFEGDTLVNRIELKGIDFKEDGNILGHKLEYNYNSHNVYIMADKQKN<br>GIKVNFKIRHNIEDGSVQLADHYQQNTPIGDGPVLLPDNHYLSTQSALSKDPNEKRDHMVLLEFVTAAGITLGMDELYKGSRS<br>HHHHHH
**20**	MQEAKIDIKSLYDSLAKKYDVQHKNSYEVIYPKGYEIKVLGNKYVKLVAMSRHKTQKHLVKIVVKSEKTIDSLDPIRQKSLLKKQD<br>EVVVTTDH**SV**MVYNDDHFFENVNAKNLKVGNYVSVYDEASDKEVIGEIASIEDLGMTDDYVYD**A**EVDDDSHAFYASNILVHN<br>SQF**A**NGTVSKGEELFTGVVPILVELDGDVNGHKFSVSGEGEGDATYGKLTLKFICTTGKLPVPWPTLVTTLTYGVQCFSRYPDH<br>MKQHDFFKSAMPEGYVQERTIFFKDDGNYKTRAEVKFEGDTLVNRIELKGIDFKEDGNILGHKLEYNYNSHNVYIMADKQKN<br>GIKVNFKIRHNIEDGSVQLADHYQQNTPIGDGPVLLPDNHYLSTQSALSKDPNEKRDHMVLLEFVTAAGITLGMDELYKGSRS<br>HHHHHH
**21**	MDEKTTGWRGGHVVEGLAGELEQLRARLEHHPQGQREPGASGGGGSSSEARDWVKRVGGKTSLSVNTYSGEVERKNINYI<br>MK**A**TVKKRMFKIKAGGKEVIVTADHSVMVKRDGKIIDVKPTEMKQTDRVVKWMLTGSHMIEFIEFEIEDLGVMEIDVYDIEV<br>DGNHNFFGNDILVHNSVYLNGTVSKGEELFTGVVPILVELDGDVNGHKFSVSGEGEGDATYGKLTLKFICTTGKLPVPWPTLVT<br>TLTYGVQCFSRYPDHMKQHDFFKSAMPEGYVQERTIFFKDDGNYKTRAEVKFEGDTLVNRIELKGIDFKEDGNILGHKLEYNYN<br>SHNVYIMADKQKNGIKVNFKIRHNIEDGSVQLADHYQQNTPIGDGPVLLPDNHYLSTQSALSKDPNEKRDHMVLLEFVTAAGI<br>TLGMDELYKGSRSHHHHHH
**22**	MDEKTTGWRGGHVVEGLAGELEQLRARLEHHPQGQREPGASGGGGSSSEARDWVKRVGGKTSLSVNTYSGEVERKNINYI<br>MKHTVKKRMFKIKAGGKEVIVTAD**A**SVMVKRDGKIIDVKPTEMKQTDRVVKWMLTGSHMIEFIEFEIEDLGVMEIDVYDIEV<br>DGNHNFFGNDILVHNSVYLNGTVSKGEELFTGVVPILVELDGDVNGHKFSVSGEGEGDATYGKLTLKFICTTGKLPVPWPTLVT

	TLTYGVQCFSRYPDHMKQHDFFKSAMPEGYVQERTIFFKDDGNYKTRAEVKFEGDTLVNRIELKGIDFKEDGNILGHKLEYNYN<br>SHNVYIMADKQKNGIKVNFKIRHNIEDGSVQLADHYQQNTPIGDGPVLLPDNHYLSTQSALSKDPNEKRDHMVLLEFVTAAGI<br>TLGMDELYKGSRSHHHHHH
**23**	MDEKTTGWRGGHVVEGLAGELEQLRARLEHHPQGQREPGASGGGGSSSEARDWVKRVGGKTSLSVNTYSGEVERKNINYI<br>MK**A**TVKKRMFKIKAGGKEVIVTAD**A**SVMVKRDGKIIDVKPTEMKQTDRVVKWMLTGSHMIEFIEFEIEDLGVMEIDVYDIEV<br>DGNHNFFGNDILVHNSVYLNGTVSKGEELFTGVVPILVELDGDVNGHKFSVSGEGEGDATYGKLTLKFICTTGKLPVPWPTLVT<br>TLTYGVQCFSRYPDHMKQHDFFKSAMPEGYVQERTIFFKDDGNYKTRAEVKFEGDTLVNRIELKGIDFKEDGNILGHKLEYNYN<br>SHNVYIMADKQKNGIKVNFKIRHNIEDGSVQLADHYQQNTPIGDGPVLLPDNHYLSTQSALSKDPNEKRDHMVLLEFVTAAGI<br>TLGMDELYKGSRSHHHHHH
**24**	MKIEEGKLVIWINGDKGYNGLAEVGKKFEKDTGIKVTVEHPDKLEEKFPQVAATGDGPDIIFWAHDRFGGYAQSGLLAEITPDK<br>AFQDKLYPFTWDAVRYNGKLIAYPIAVEALSLIYNKDLLPNPPKTWEEIPALDKELKAKGKSALMFNLQEPYFTWPLIAADGGYA<br>FKYENGKYDIKDVGVDNAGAKAGLTFLVDLIKNKHMNADTDYSIAEAAFNKGETAMTINGPWAWSNIDTSKVNYGVTVLPTF<br>KGQPSKPFVGVLSAGINAASPNKELAKEFLENYLLTDEGLEAVNKDKPLGAVALKSYEEELAKDPRIAATMENAQKGEIMPNIP<br>QMSAFWYAVRTAVINAASGRQTVDEALKDAQTNSSSNNNNNNNNNNLGIEGRGTLEASILPEEWVPLIKNGKVKIFRIGDFV<br>DGLMKANQGKVKKTGDTEVLEVAGIHAFSFDRKSKKARVMAVKAVIRHRYSGNVYRIVLNSGRKITITEGHSLFVYRNGDLVE<br>ATGEDVKIGDLLAVPRSVNLPEKRERLNIVELLLNLSPEETEDIILTIPVKGRKNFFKGMLRTLRWIFGEEKRVRTASRYLRHLENL<br>GYIRLRKIGYDIIDKEGLEKYRTLYEKLVDVVRYNGNKREYLVEFNAVRDVISLMPEEELKEWRIGTRNGFRMGTFVDIDEDFAKL<br>LGYYVSEGSARKWKNQTGGWSYTVRLYNENDEVLDDMEHLAKKFFGKVKRGKNYVEIPKKMAYIIFESLCGTLAENKRVPEVI<br>FTSSKGVRWAFLEGYFIGDGDVHPSKRVRLSTKSELLVNGLVLLLNSLGVSAIKLGYDSGVYRVYVNEELKFTEYRKKKNVYHSHI<br>VPKDILKETFGKVFQKNISYKKFRELVENGKLDREKAKRIEWLLNGDIVLDRVVEIKREYYDGYVYDLSVDEDENFLAGFGSLYAH<br>NSGLNSAFGSMSVADLGSLTRLEDKIRLLQEDLESERELRNRIERERADLSVQLIALTDRLEDAEGTTDSQIESNRKREAELQKLR<br>KLLEESQLENEDAMNVLRKKHQDACLDYAEQIEQLQKKNSKIDRERQRLQHEVIELTATIDQLQKDKHLAEKAAERFEAQTIELS<br>NKVEDLNRHVNDLAQQRQRLQAENNDLLKEIHDQKVQLDNLQHVKYQLAQQLEEARRPAGKLGTGRRFTTS
**25**	MKIEEGKLVIWINGDKGYNGLAEVGKKFEKDTGIKVTVEHPDKLEEKFPQVAATGDGPDIIFWAHDRFGGYAQSGLLAEITPDK<br>AFQDKLYPFTWDAVRYNGKLIAYPIAVEALSLIYNKDLLPNPPKTWEEIPALDKELKAKGKSALMFNLQEPYFTWPLIAADGGYA<br>FKYENGKYDIKDVGVDNAGAKAGLTFLVDLIKNKHMNADTDYSIAEAAFNKGETAMTINGPWAWSNIDTSKVNYGVTVLPTF<br>KGQPSKPFVGVLSAGINAASPNKELAKEFLENYLLTDEGLEAVNKDKPLGAVALKSYEEELAKDPRIAATMENAQKGEIMPNIP<br>QMSAFWYAVRTAVINAASGRQTVDEALKDAQTNSSSNNNNNNNNNNLGIEGRGTLEASILPEEWVPLIKNGKVKIFRIGDFV<br>DGLMKANQGKVKKTGDTEVLEVAGIHAFSFDRKSKKARVMAVKAVIRHRYSGNVYRIVLNSGRKITITEGHSLFVYRNGDLVE<br>ATGEDVKIGDLLAVPRSVNLPEKRERLNIVELLLNLSPEETEDIILTIPVKGRKNFFKGMLRTLRWIFGEEKRVRTASRYLRHLENL<br>GYIRLRKIGYDIIDKEGLEKYRTLYEKLVDVVRYNGNKREYLVEFNAVRDVISLMPEEELKEWRIGTRNGFRMGTFVDIDEDFAKL<br>LGYYVSEGSARKWKNQTGGWSYTVRLYNENDEVLDDMEHLAKKFFGKVKRGKNYVEIPKKMAYIIFESLCGTLAENKRVPEVI<br>FTSSKGVRWAFLEGYFIGDGDVHPSKRVRLSTKSELLVNGLVLLLNSLGVSAIKLGYDSGVYRVYVNEELKFTEYRKKKNVYHSHI<br>VPKDILKETFGKVFQKNISYKKFRELVENGKLDREKAKRIEWLLNGDIVLDRVVEIKREYYDGYVYDLSVDEDENFLAGFGSLYAH<br>NSGLNSAFGSMSVADLGSLTRLEDKIRLLQEDLESERELRNRIERERADLSVQLIALTDRLEDAEGTTDSQIESNRKREAELQKLR<br>KLLEESQLENEDAMNVLRKKHQDACLDYAEQIEQLQKKNSKIDRERQRLQHEVIELTATIDQLQKDKHLAEKAAERFEAQTIELS<br>NKVEDLNRHVNDLAQQRQRLQAENNDLLKEIHDQKVQLDNLQHVKYQLAQQLEEARRPAGKLGTGRRFTTS
**26**	MKIEEGKLVIWINGDKGYNGLAEVGKKFEKDTGIKVTVEHPDKLEEKFPQVAATGDGPDIIFWAHDRFGGYAQSGLLAEITPDK<br>AFQDKLYPFTWDAVRYNGKLIAYPIAVEALSLIYNKDLLPNPPKTWEEIPALDKELKAKGKSALMFNLQEPYFTWPLIAADGGYA<br>FKYENGKYDIKDVGVDNAGAKAGLTFLVDLIKNKHMNADTDYSIAEAAFNKGETAMTINGPWAWSNIDTSKVNYGVTVLPTF<br>KGQPSKPFVGVLSAGINAASPNKELAKEFLENYLLTDEGLEAVNKDKPLGAVALKSYEEELAKDPRIAATMENAQKGEIMPNIP<br>QMSAFWYAVRTAVINAASGRQTVDEALKDAQTNSSSNNNNNNNNNNLGIEGRGTLEASILPEEWVPLIKNGKVKIFRIGDFV<br>DGLMKANQGKVKKTGDTEVLEVAGIHAFSFDRKSKKARVMAVKAVIR**A**RYSGNVYRIVLNSGRKITITEGASLFVYRNGDLVEA<br>TGEDVKIGDLLAVPRSVNLPEKRERLNIVELLLNLSPEETEDIILTIPVKGRKNFFKGMLRTLRWIFGEEKRVRTASRYLRHLENLGY<br>IRLRKIGYDIIDKEGLEKYRTLYEKLVDVVRYNGNKREYLVEFNAVRDVISLMPEEELKEWRIGTRNGFRMGTFVDIDEDFAKLLG<br>YYVSEGSARKWKNQTGGWSYTVRLYNENDEVLDDMEHLAKKFFGKVKRGKNYVEIPKKMAYIIFESLCGTLAENKRVPEVIFTS<br>SKGVRWAFLEGYFIGDGDVHPSKRVRLSTKSELLVNGLVLLLNSLGVSAIKLGYDSGVYRVYVNEELKFTEYRKKKNVYHSHIVPK<br>DILKETFGKVFQKNISYKKFRELVENGKLDREKAKRIEWLLNGDIVLDRVVEIKREYYDGYVYDLSVDEDENFLAGFGSLYAHNSG<br>LNSAFGSMSVADLGSLTRLEDKIRLLQEDLESERELRNRIERERADLSVQLIALTDRLEDAEGTTDSQIESNRKREAELQKLRKLLE<br>ESQLENEDAMNVLRKKHQDACLDYAEQIEQLQKKNSKIDRERQRLQHEVIELTATIDQLQKDKHLAEKAAERFEAQTIELSNKV<br>EDLNRHVNDLAQQRQRLQAENNDLLKEIHDQKVQLDNLQHVKYQLAQQLEEARRPAGKLGTGRRFTTS
**27**	MKTEEGKLVIWINGDKGYNGLAEVGKKFEKDTGIKVTVEHPDKLEEKFPQVAATGDGPDIIFWAHDRFGGYAQSGLLAEITPD<br>KAFQDKLYPFTWDAVRYNGKLIAYPIAVEALSLIYNKDLLPNPPKTWEEIPALDKELKAKGKSALMFNLQEPYFTWPLIAADGGY<br>AFKYENGKYDIKDVGVDNAGAKAGLTFLVDLIKNKHMNADTDYSIAEAAFNKGETAMTINGPWAWSNIDTSKVNYGVTVLP<br>TFKGQPSKPFVGVLSAGINAASPNKELAKEFLENYLLTDEGLEAVNKDKPLGAVALKSYEEELAKDPRIAATMENAQKGEIMPNI<br>PQMSAFWYAVRTAVINAASGRQTVDEALKDAQTNSSSNNNNNNNNNNLGIEGRGTLEEFVIYADSVVKDAKVIIKEDGKIKEI<br>KIEDLFKKVDYTIGDKEYCILNNVETLTIEDTKLVWRKVPYIMRHRTNKKIYRVKVKDRYVDITEDHSIIGVKNNKLVELKPTEIKD<br>DETKLIILNKDLKSYNFASVEEINCIKYSDYVYDIEVENTHRFFANGILVHNTDGFYGTGMSDKIIHLTDDSFDTDVLKADGAILVD<br>FWAEWCGPCKMIAPILDEIADEYQGKLTVAKLNIDQNPGTAPKYGIRGIPTLLLFKNGEVAATKVGALSKGQLKEFLDANLAHH<br>HHHH
**28**	MKTEEGKLVIWINGDKGYNGLAEVGKKFEKDTGIKVTVEHPDKLEEKFPQVAATGDGPDIIFWAHDRFGGYAQSGLLAEITPD<br>KAFQDKLYPFTWDAVRYNGKLIAYPIAVEALSLIYNKDLLPNPPKTWEEIPALDKELKAKGKSALMFNLQEPYFTWPLIAADGGY

	AFKYENGKYDIKDVGVDNAGAKAGLTFLVDLIKNKHMNADTDYSIAEAAFNKGETAMTINGPWAWSNIDTSKVNYGVTVLP<br>TFKGQPSKPFVGVLSAGINAASPNKELAKEFLENYLLTDEGLEAVNKDKPLGAVALKSYEEELAKDPRIAATMENAQKGEIMPNI<br>PQMSAFWYAVRTAVINAASGRQTVDEALKDAQTNSSSNNNNNNNNNNNLGIEGRGTLEEFVIYADSVVKDAKVIIKEDGKIKEI<br>KIEDLFKKVDYTIGDKEYCILNNVETLTIEDTKLVWRKVPYIMRARTNKKIYRVKVKDRYVDITEDHSIIGVKNNKLVELKPTEIKDD<br>ETKLIILNKDLKSYNFASVEEINCIKYSDYVYDIEVENTHRFFANGILVHNTDGFYGTGMSDKIIHLTDDSFDTDVLKADGAILVDF<br>WAEWCGPCKMIAPILDEIADEYQGKLTVAKLNIDQNPGTAPKYGIRGIPTLLLFKNGEVAATKVGALSKGQLKEFLDANLAHH<br>HHHH
**29**	MKTEEGKLVIWINGDKGYNGLAEVGKKFEKDTGIKVTVEHPDKLEEKFPQVAATGDGPDIIFWAHDRFGGYAQSGLLAEITPD<br>KAFQDKLYPFTWDAVRYNGKLIAYPIAVEALSLIYNKDLLPNPPKTWEEIPALDKELKAKGKSALMFNLQEPYFTWPLIAADGGY<br>AFKYENGKYDIKDVGVDNAGAKAGLTFLVDLIKNKHMNADTDYSIAEAAFNKGETAMTINGPWAWSNIDTSKVNYGVTVLP<br>TFKGQPSKPFVGVLSAGINAASPNKELAKEFLENYLLTDEGLEAVNKDKPLGAVALKSYEEELAKDPRIAATMENAQKGEIMPNI<br>PQMSAFWYAVRTAVINAASGRQTVDEALKDAQTNSSSNNNNNNNNNNNLGIEGRGTLEEFVIYADSVVKDAKVIIKEDGKIKEI<br>KIEDLFKKVDYTIGDKEYCILNNVETLTIEDTKLVWRKVPYIMRHRTNKKIYRVKVKDRYVDITEDASIIGVKNNKLVELKPTEIKDD<br>ETKLIILNKDLKSYNFASVEEINCIKYSDYVYDIEVENTHRFFANGILVHNTDGFYGTGMSDKIIHLTDDSFDTDVLKADGAILVDF<br>WAEWCGPCKMIAPILDEIADEYQGKLTVAKLNIDQNPGTAPKYGIRGIPTLLLFKNGEVAATKVGALSKGQLKEFLDANLAHH<br>HHHH
**30**	MKTEFSGDTDAVHGKTHVFIRSIKNGSHMQEAKIDIKSLYDSLAKKYDVQHKNSYEVIYPKGYEIKVLGNKYVKLVAMSRHKTQ<br>KHLVKIVVKSEKTIDSLDPIRQKSLLKKQDEVVVTTDHICMVYNDDHFFENVNAKNLKVGNYVSVYDEASDKEVIGEIASIEDLG<br>MTDDYVYDCEVDDDSHAFYASNILVHASQFCNGTKLGGCFSGDTLVALTDGRSVSFEQLVEEEKQGKQNFCYTIRHDGSIGVE<br>KIINARKTKTNAKVIKVTLDNGESIICTPDHKFMLRDGSYKCAMDLTLDDSLMPLHRKISTTEDSGHMEAVLNYNHRIVNIEAVS<br>ETIDVYDIEVPHTHNFALASTGMKIEEGKLTNPGVSAWQVNTAYTAGQLVTYNGKTYKCLQPHTSLAGWEPSNVPALWQLQ
**31**	MKTEFSGDTDAVHGKTHVFIRSIKNGSHMQEAKIDIKSLYDSLAKKYDVQHKNSYEVIYPKGYEIKVLGNKYVKLVAMSRHKTQ<br>KHLVKIVVKSEKTIDSLDPIRQKSLLKKQDEVVVTTDHIAMVYNDDHFFENVNAKNLKVGNYVSVYDEASDKEVIGEIASIEDLG<br>MTDDYVYDAEVDDDSHAFYASNILVHASQFCNGTKLGGCFSGDTLVALTDGRSVSFEQLVEEEKQGKQNFCYTIRHDGSIGVE<br>KIINARKTKTNAKVIKVTLDNGESIICTPDHKFMLRDGSYKCAMDLTLDDSLMPLHRKISTTEDSGHMEAVLNYNHRIVNIEAVS<br>ETIDVYDIEVPHTHNFALASTGMKIEEGKLTNPGVSAWQVNTAYTAGQLVTYNGKTYKCLQPHTSLAGWEPSNVPALWQLQ
**32**	MKTEEGKLVIWINGDKGYNGLAEVGKKFEKDTGIKVTVEHPDKLEEKFPQVAATGDGPDIIFWAHDRFGGYAQSGLLAEITPD<br>KAFQDKLYPFTWDAVRYNGKLIAYPIAVEALSLIYNKDLLPNPPKTWEEIPALDKELKAKGKSALMFNLQEPYFTWPLIAADGGY<br>AFKYENGKYDIKDVGVDNAGAKAGLTFLVDLIKNKHMNADTDYSIAEAAFNKGETAMTINGPWAWSNIDTSKVNYGVTVLP<br>TFKGQPSKPFVGVLSAGINAASPNKELAKEFLENYLLTDEGLEAVNKDKPLGAVALKSYEEELAKDPRIAATMENAQKGEIMPNI<br>PQMSAFWYAVRTAVINAASGRQTVDEALKDAQTNSSSNNNNNNNNNNNLGIEGRGTLEEFSGDTDSVHGKTHVFIRSIKNGS<br>HHHHHH
**33**	MQEAKIDIKSLYDSLAKKYDVQHKNSYEVIYPKGYEIKVLGNKYVKLVAMSRHKTQKHLVKIVVKSEKTIDSLDPIRQKSLLKKQD<br>EVVVTTDHICMVYNDDHFFENVNAKNLKVGNYVSVYDEASDKEVIGEIASIEDLGMTDDYVYDCEVDDDSHAFYASNILVHNS<br>QFCNGTGMSDKIIHLTDDSFDTDVLKADGAILVDFWAEWCGPCKMIAPILDEIADEYQGKLTVAKLNIDQNPGTAPKYGIRGIP<br>TLLLFKNGEVAATKVGALSKGQLKEFLDANLA

Supporting Figures

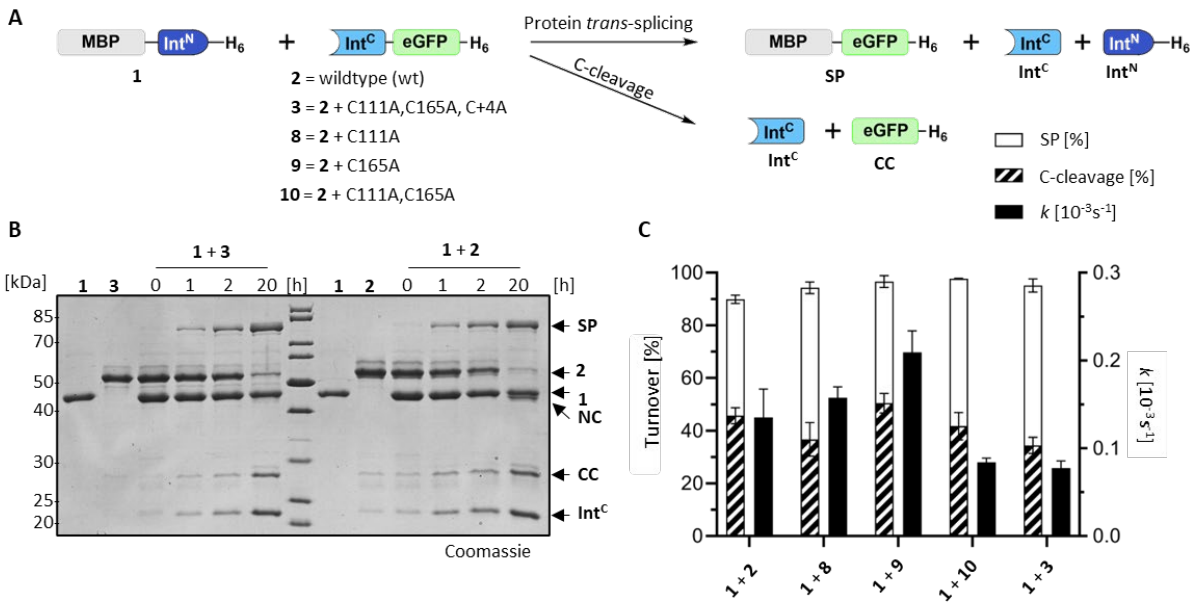

Figure S1 Protein trans-splicing activity of the split PolB16 intein with removed, non-conserved cysteine residues. **A)** Schematic reaction overview of the two precursor proteins MBP-Int^{N}-His$_6$ (**1**) and Int^{C}-eGFP-His$_6$ (**2**, **3**, **8**-**10**), which form the desired splice product (**SP**) and the byproducts Int^{C} and Int^{N}. C-Cleavage forms the side product C-Cleavage (**CC**) next to Int^{C}. **B)** SDS-PAGE of the splice assays with the native Int^{N}-precursor (**1**;10 µM) in excess towards the cysteine-free (**3**) or the wildtype (**2**) Int^{C}-precursor (5 µM) at 25°C and pH 7. **C)** Yields of the total turnover as the sum of the **SP** (white) and **CC** (diagonally striped). Integrated into the same diagram is splicing rate (black; see y-axis on the right hand side) for the indicated split intein combinations (n=3; error bars represent standard deviations).

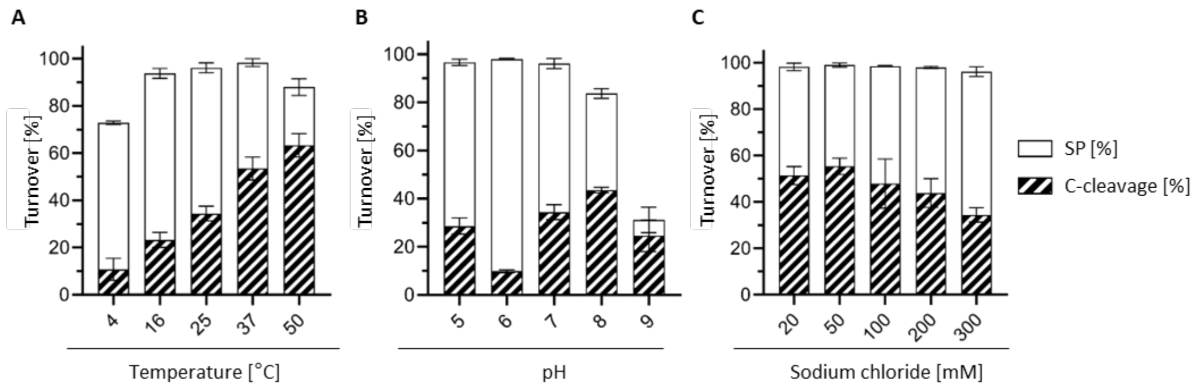

Figure S2 Protein trans-splicing activity of the split PolB16 intein under various conditions **A-C)** Yields of the total turnover as the sum of the **SP** (white) and **CC** (diagonally striped). Integrated into the same diagram is splicing rate (black; see y-axis on the right hand side) for the PB16 intein (**1**+**3**) at different temperatures (**A,** n=3), at different pH-values (**B**, n=3) and at different sodium chloride concentrations (**C**, n=3). Error bars represent standard deviations.

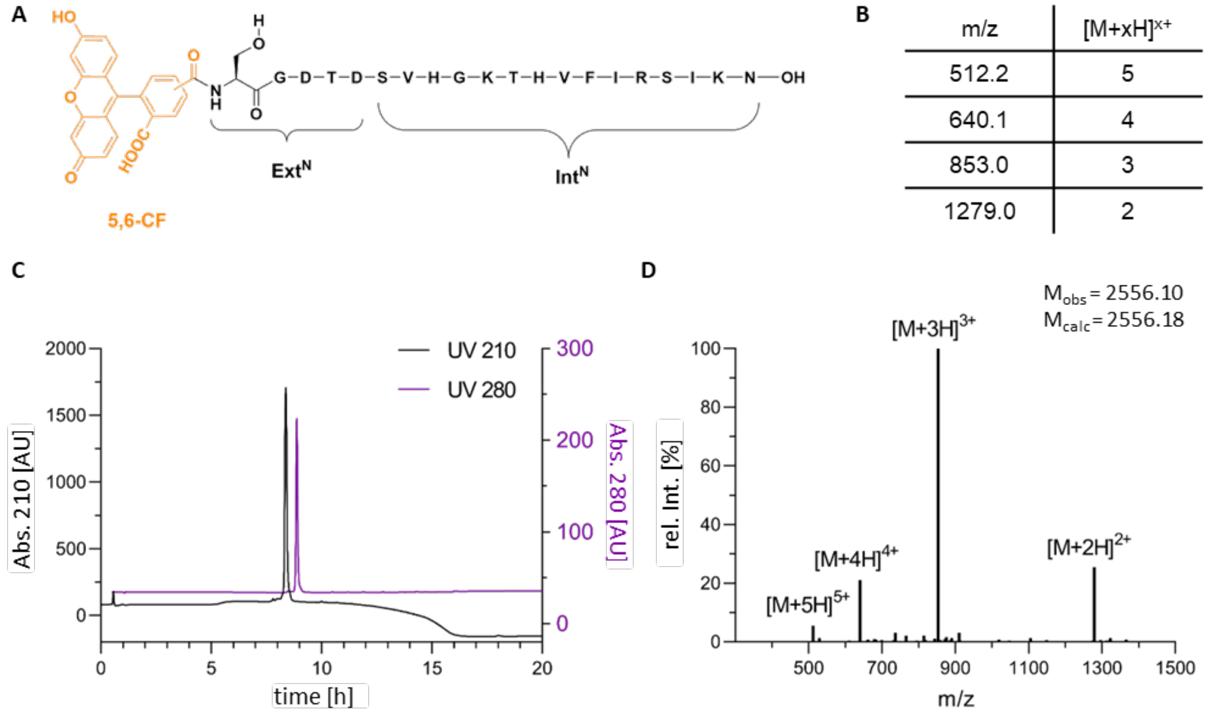

Figure S3 Solid-phase peptide synthesis of the synthetic PolB16 Int^{N} precursor peptide. **A)** Scheme of the synthesized peptide 5(6)-CF-SGDTDSVHGKTHVFIRSIKN-OH (**4**). **B)** Observed m/z values of the purified peptide. **C)** UV traces at 210 nm (black) and 280 nm (blue) of the purified peptide. **D)** Extracted ion chromatogram of the purified peptide.

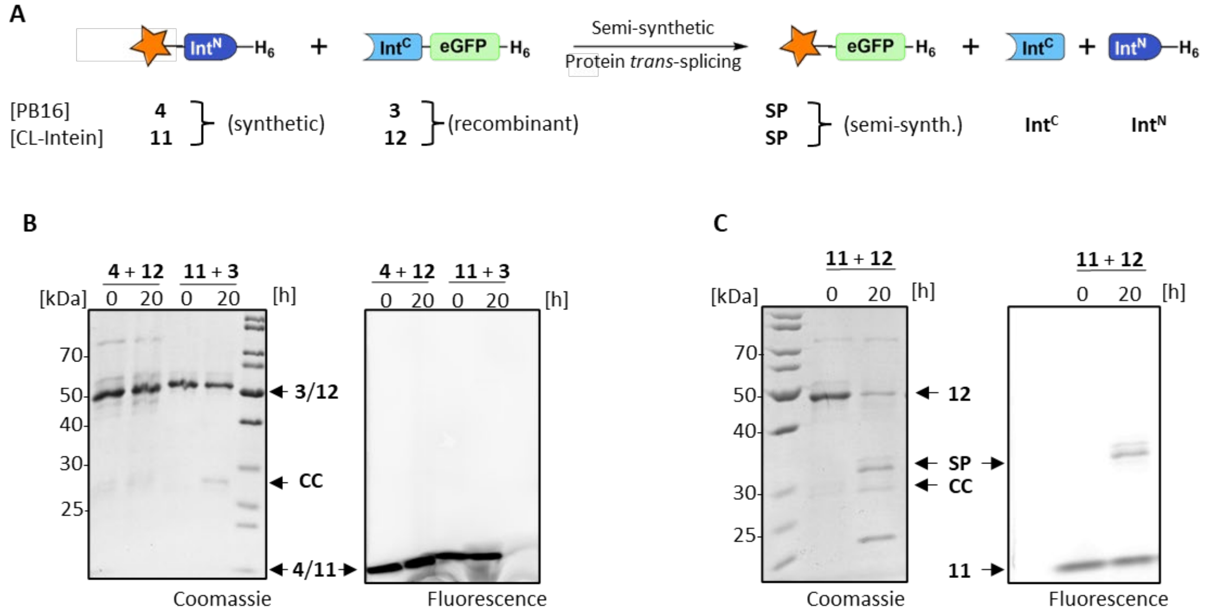

Figure S4 Orthogonality of the split PolB16 and CL inteins. **A)** Schematic reaction overview of semi-synthetic protein trans-splicing of the two cysteine-less split inteins PolB16 (this work) and the previously reported CL.[13] **B)** SDS-PAGE of the semi-synthetic cross splice assays with the recombinant Int^{C}-precursors (each 5 µM) and the synthetic Int^{N}-precursors (each 15 µM) at 25°C and pH 7. Additional Fluorescence-scan at 495 nm of the SDS-PAGE gel. No splice product formation was observed, showing the orthogonality of the two split inteins. **C)** SDS-PAGE of the semi-synthetic assay with the recombinant Int^{C}-precursor (5 µM) and the synthetic Int^{N}-precursors (15 µM) at 25°C and pH 7.

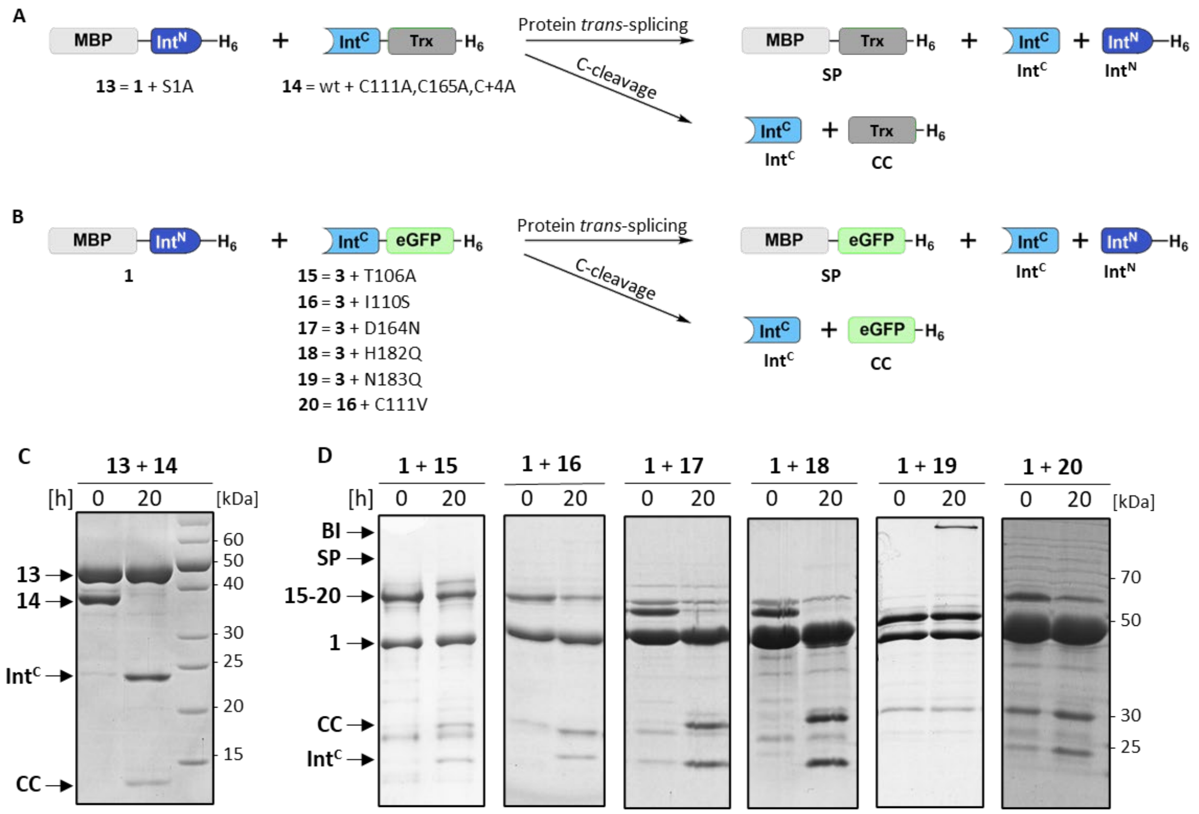

Figure S5 Mutational analysis of the split PolB16 intein. **A-B)** Schemes of the reactions. **C-D)** SDS-PAGE analyses (Coomassie-stained) of the indicated protein trans-splicing reactions. SP = splice product. BI = branched intermediate.

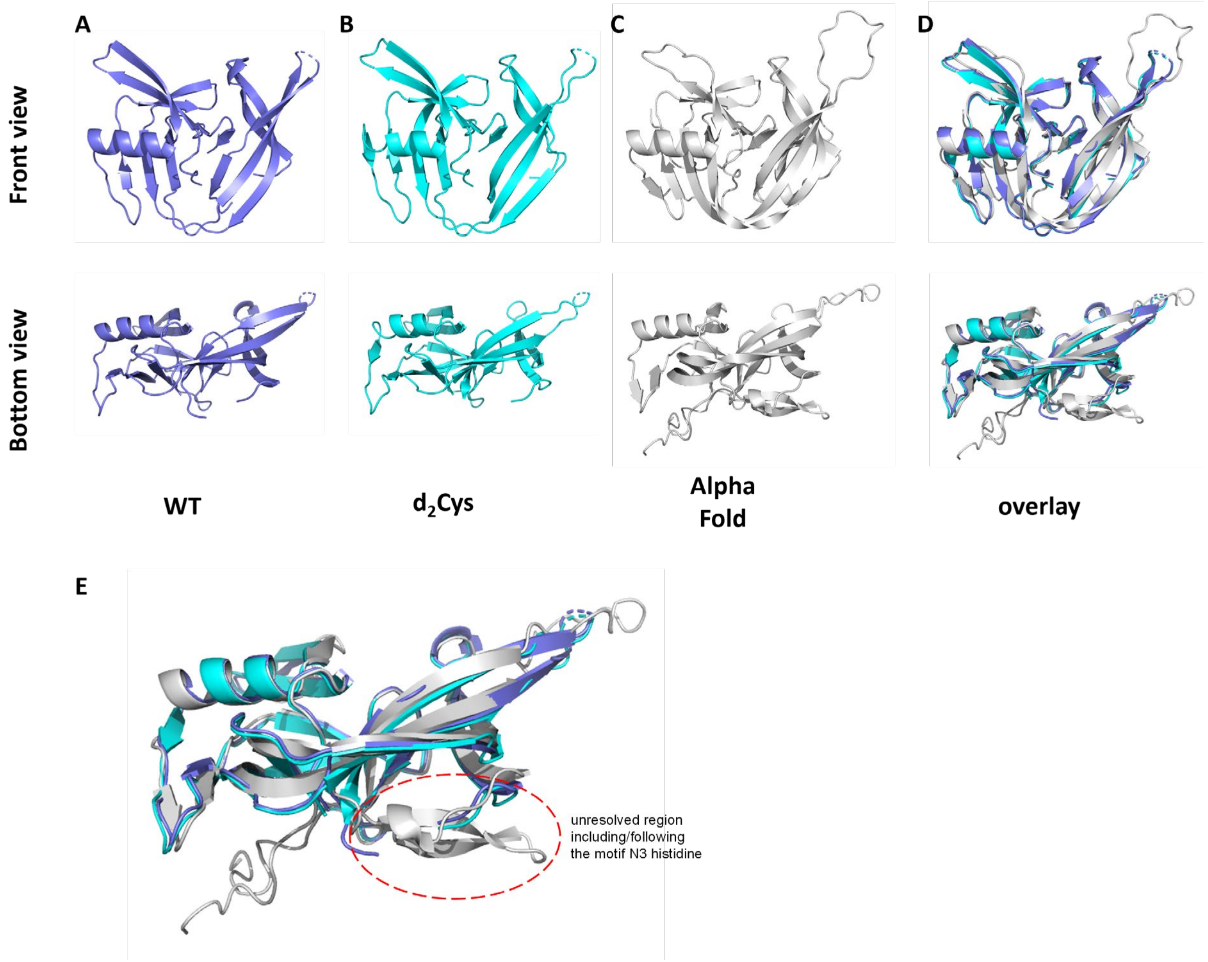

Figure S6 Structural analysis of the PolB16 intein reveals unfolded region around motif N3 histidine. Shown are crystal structures of the wild type (A) and the cysteine-free version d_2Cys (B) of the PolB16 intein. Panel C) shows the structural model calculated by AlphaFold[15] for comparison. D) Overlay of the three structures. E) Magnification of the overlayed structures shown in D) (bottom view) to highlight the region unresolved in the experimental crystal structures and the comparison to the AlphaFold model, which places the motif N3 histidine in the expected position according to experimentally determined intein structures.

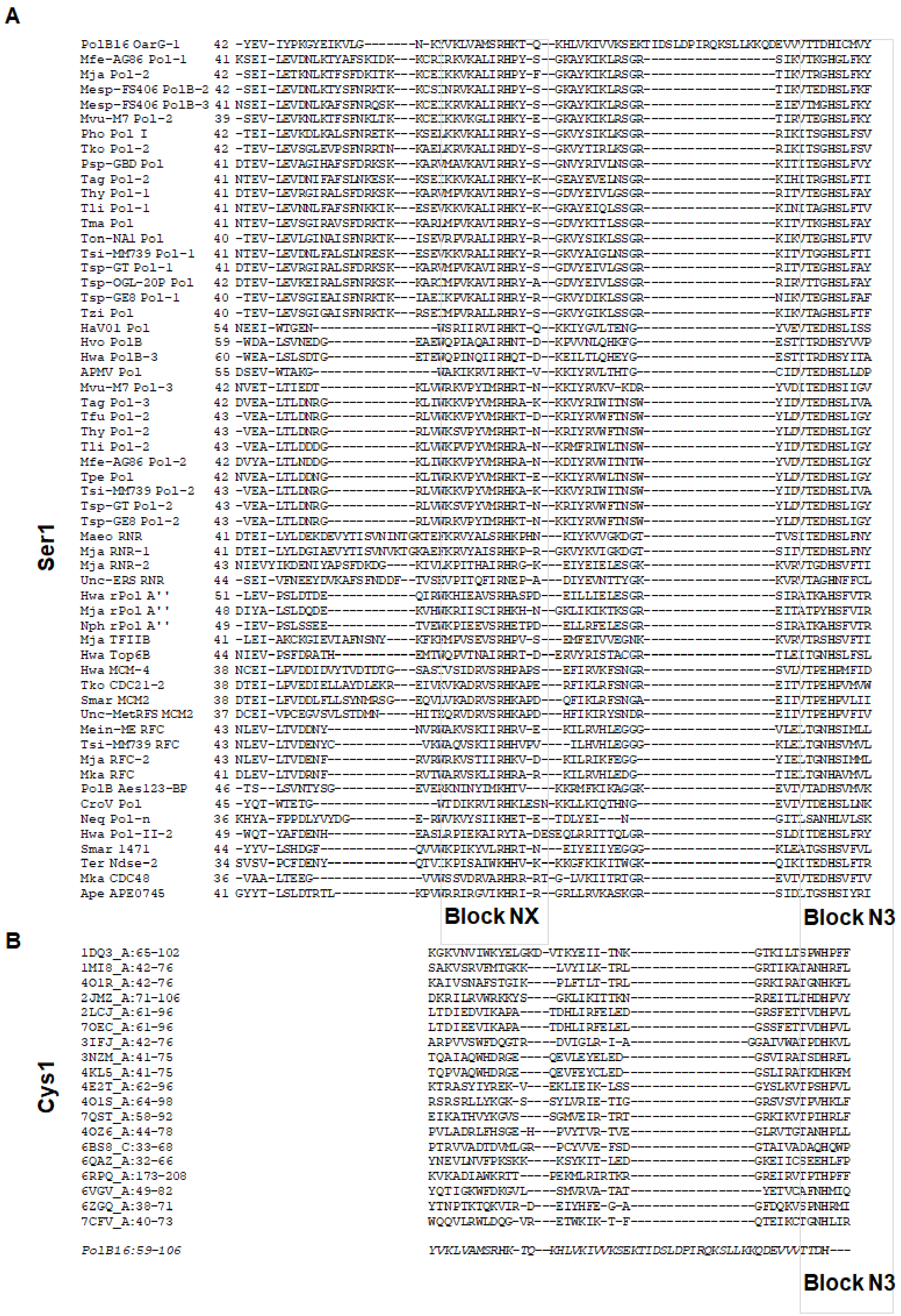

Figure continued >>>

>>> Figure continued

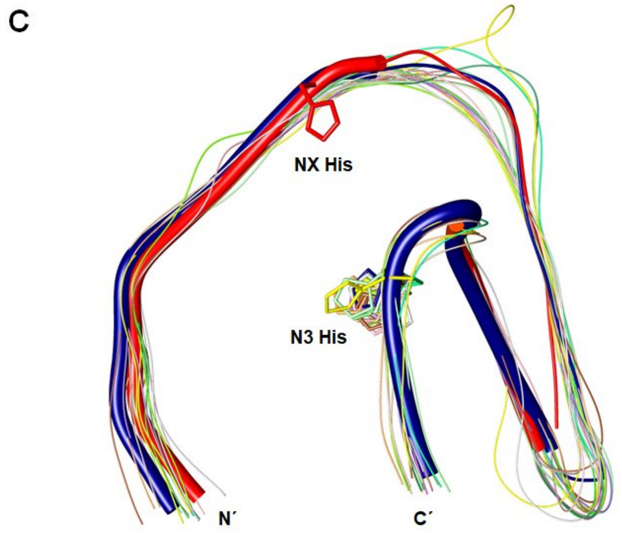

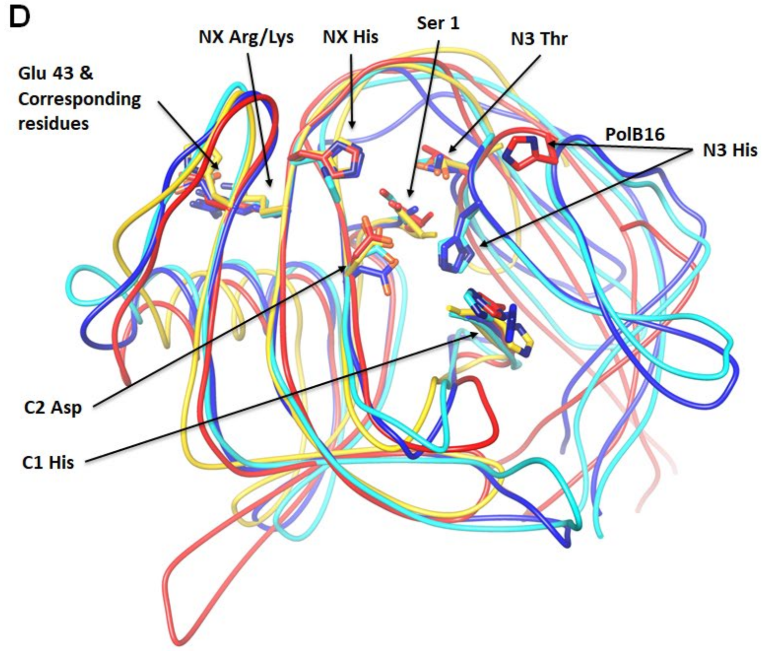

Figure S7 Sequence and structural alignments of intein. A) Multiple sequence alignments (MSA) of protein sequences of the NX (or corresponding region) to N3 motifs with Ser1/Ser+1|Thr+1 inteins from InBase.[4] B) Similar MSA as in A) but using Cys1 inteins of known structure. The corresponding PolB16 sequence shown in italics is for reference and is not part of this alignment. These alignments were used to create the logo motifs representations shown in Figure 7. C) Structural superposition of the Cys1 structures listed in B using Cα atoms of the segments depicted by thick coils. PolB16 (red), which is not included in B, was overlayed on a representative Cys1 structure, 7OEC (blue). Motif NX of PolB16 and the corresponding Cys1 inteins regions are on the left towards the N' end, with the catalytic PolB16 His side chain shown. Motif N3 regions are on the right towards the C' end, with the catalytic His side chains shown. D) Structural overlay of four available Ser1 intein structures using corresponding Cα atoms. PolB16 is shown in red. Other inteins are Mja-TFIIB mini-intein (blue, PDB 5O9I), Neq Pol-n/Pol-c complex, (yellow, PDB 5OXX), and Tko Pol-2 (cyan, PDB 2CW7). The UCSF-Chimera package[16] was used for structures overlay and for preparing panels C and D.

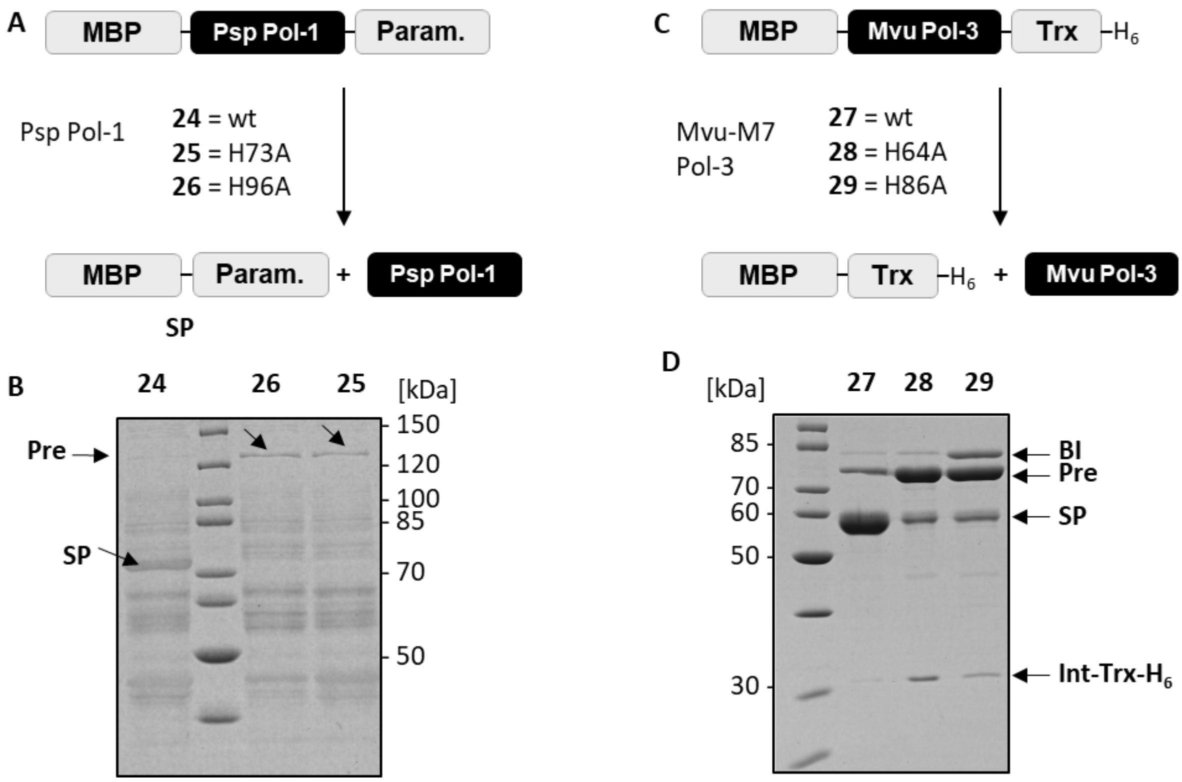

Figure S8 Catalytic histidine mutations in two Ser1 cis-inteins. A) Reaction of the cis-splicing Psp GBD-Pol1 precursor[14] and its mutants. His73 and His96 are the motif NX and motif N3 histidines of this intein, respectively. Param. = Paramyosin. B) Analysis of the reactions shown in A). Following recombinant precursor expression, MBP-containing proteins were purified on an amylose resin and analyzed on the presented Coomassie-stained SDS-PAGE gel. A high background of contaminating proteins can be observed, yet splice product (SP = MBP-Param.; Mcalc = 70.945 kDa) and unspliced precursor proteins (pre; Mcalc 133.106 kDa) are clearly visible. A plasmid with the DNA for expression of the wildtype precursor protein was kindly provided by Francine Perler (New England Biolabs). C) Reaction of the cis-splicing Mvu-M7-Pol3 intein and its mutants. His64 and His86 are the motif NX and motif N3 histidines of this intein, respectively. Recombinant precursor proteins were expressed in E. coli and from each cell extract His_6-tagged proteins were purified by Ni-NTA affinity chromatography. D) Analysis was performed on a Coomassie-stained SDS-PAGE gel as shown. Unspliced precursor (Mcalc = 75.769 kDa) as well as splice product (Mcalc = 57.118 kDa) can be observed. For the His64 and His86 mutants Intein-Trx-H6 (Mcalc = 32.061 kDa) as a by-product of N-terminal cleavage is detectable. BI = branched intermediated; SP = splice product (MBP-Trx-His_6)

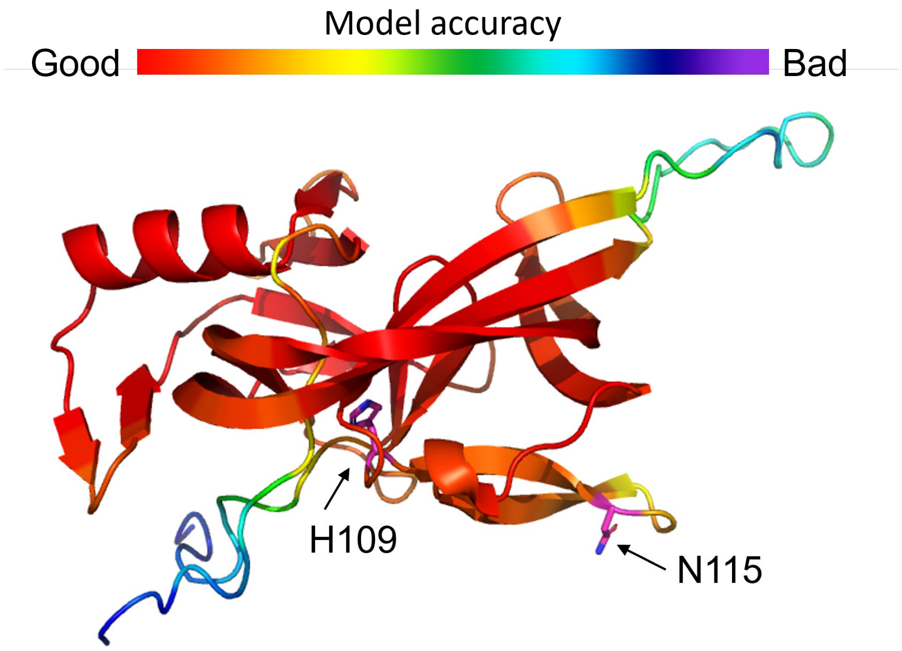

Figure S9 Calculated accuracy of the AlphaFold[15] model of the wildtype PolB-16 intein. The sequences of the Int^{N} and Int^{C} precursors were treated as a single polypeptide chain to generate this model. Another representation of the same AlphaFold model is shown in Figure S6.

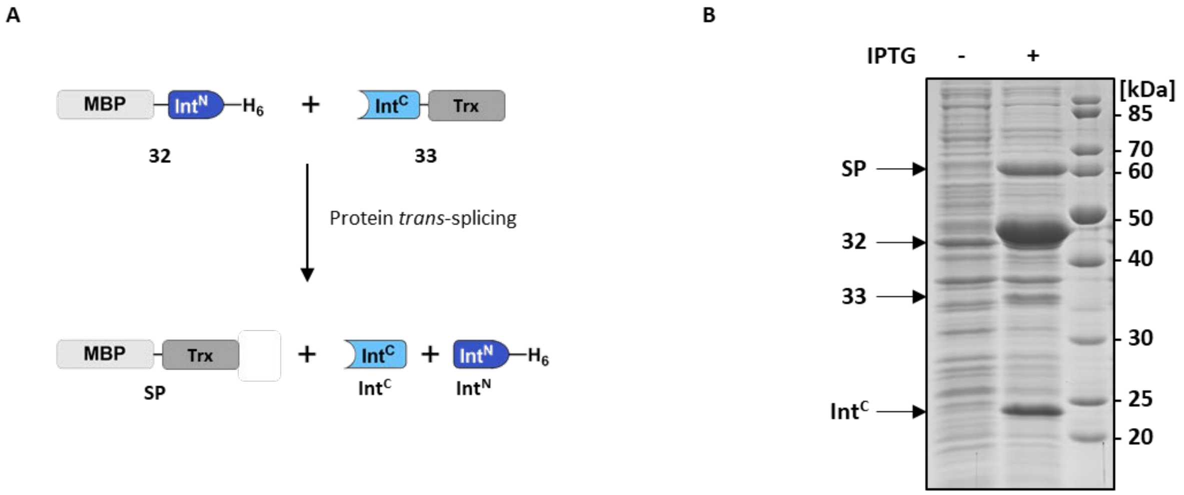

Figure S10 In vivo co-expression of PolB16 split intein precursors in *E. coli*. MBP-Int^{N}-H$_{6}$ (**32**) and Int^{C}-Trx (**33**) we co-expressed from a bicistronic arrangement on a single plasmid (pTP022) in *E. coli* BL21(DE3) cells. To this end, the sequence between the genes encoding for **32** and **33** comprised a stop codon (TAA) to terminate translation of the gene encoding **32**, a ribosomal binding site (AGGAGG) and the start codon of the gene encoding **32** embedded in an NdeI restriction site as follows: 5'-TAAGCTTTAAGGAGGATCCCATATG-3'. Cells were grown in LB medium to an $OD_{(600)}$=0.6 and an aliquot (-) removed for analysis. The culture was then induced with IPTG (0.4 mM) and after 4h at 37°C another aliquot (+) was removed for analysis. The removed cells were spun down, lysed in the denaturing conditions of SDS-PAGE buffer containing SDS and β-mercaptoethanol (10 min; 95°C) to rule out any protein trans-splicing prior to cell lysis, and analyzed on an SDS-PAGE gel stained with Coomassie brilliant blue as show. Formation of the splice product (**SP**) confirms split intein precursor recognition and protein trans-splicing took place in the complex environment of the *E. coli* cell. Note that the Int^{N} precursor (**32**) is much stronger expressed than the Int^{C} precursor (**33**) due to the operon arrangement of the two genes with the Int^{C} precursor being encoded by the second gene. Calculated molecular weights are: 46.3 kDa (**32**), 31.9 kDa (**33**), 56.2 kDa (**SP**), 2.7 kDa (**Int^{N}**), 19.3 kDa (**Int^{C}**).

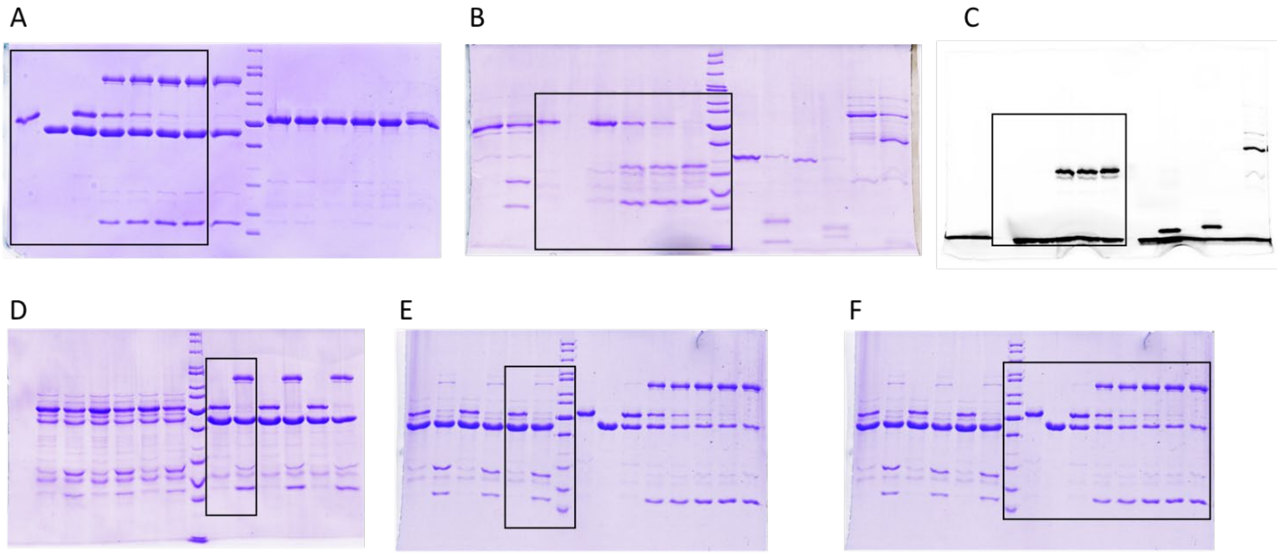

Figure S10: Unprocessed images of SDS gels, part I. The black frames indicate the sections used for the figures A) Fig. 3B. B) Fig. 4B. C) Fig. 4C. D+E) Fig. 5B. F) Fig. 8A, respectively.

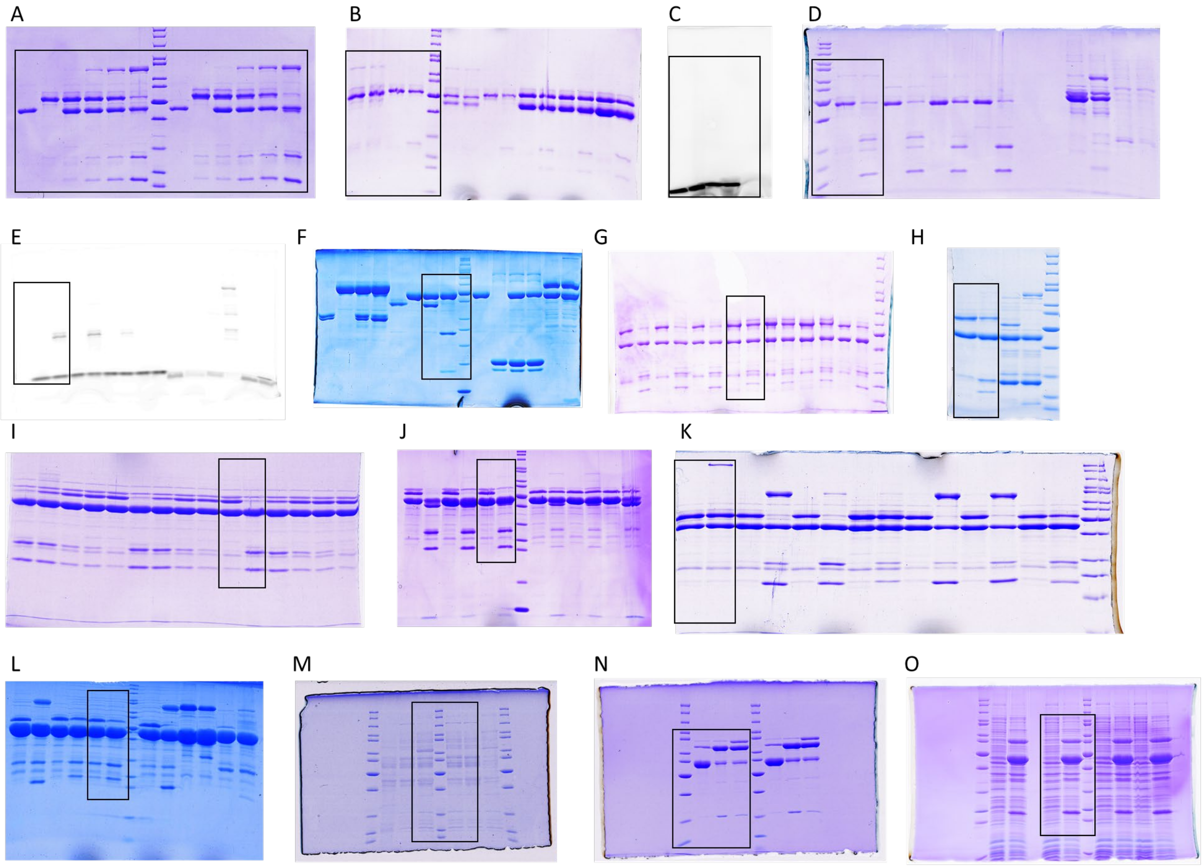

Figure S11: Unprocessed images of SDS gels, part II. The black frames indicate the sections used for the figures A) Fig. S1B. B+C) Fig. S4B. D+E) Fig. S4C. F+G) Fig. S5C. H+I+J+K+L) Fig. S5D. M) Fig. S8B. N) Fig. S8D). O) Fig. S10B, respectively.

Supporting References

1. S. Hoffmann, T. M. E. Terhorst, R. K. Singh, D. Kummel, S. Pietrokovski and H. D. Mootz, *Chembiochem*, 2021, **22**, 364-373.
2. W. Shi, C. D. Moon, S. C. Leahy, D. Kang, J. Froula, S. Kittelmann, C. Fan, S. Deutsch, D. Gagic, H. Seedorf, W. J. Kelly, R. Atua, C. Sang, P. Soni, D. Li, C. S. Pinares-Patino, J. C. McEwan, P. H. Janssen, F. Chen, A. Visel, Z. Wang, G. T. Attwood and E. M. Rubin, *Genome Res*, 2014, **24**, 1517-1525.
3. M. C. Frith, N. F. Saunders, B. Kobe and T. L. Bailey, *PLoS Comput Biol*, 2008, **4**, e1000071.
4. F. B. Perler, *Nucleic Acids Res*, 2002, **30**, 383-384.
5. G. Volkmann and X. Q. Liu, *Febs J*, 2011, **278**, 3431-3446.
6. U. Mueller, N. Darowski, M. R. Fuchs, R. Forster, M. Hellmig, K. S. Paithankar, S. Puhringer, M. Steffien, G. Zocher and M. S. Weiss, *J Synchrotron Radiat*, 2012, **19**, 442-449.
7. M. Krug, M. S. Weiss, U. Heinemann and U. Mueller, *J Appl Crystallogr*, 2012, **45**, 568-572.
8. T. C. Terwilliger, P. D. Adams, R. J. Read, A. J. McCoy, N. W. Moriarty, R. W. Grosse-Kunstleve, P. V. Afonine, P. H. Zwart and L. W. Hung, *Acta Crystallogr D Biol Crystallogr*, 2009, **65**, 582-601.
9. T. C. Terwilliger, R. W. Grosse-Kunstleve, P. V. Afonine, N. W. Moriarty, P. H. Zwart, L. W. Hung, R. J. Read and P. D. Adams, *Acta Crystallogr D Biol Crystallogr*, 2008, **64**, 61-69.
10. P. Emsley, B. Lohkamp, W. G. Scott and K. Cowtan, *Acta Crystallogr D Biol Crystallogr*, 2010, **66**, 486-501.
11. P. D. Adams, P. V. Afonine, G. Bunkoczi, V. B. Chen, I. W. Davis, N. Echols, J. J. Headd, L. W. Hung, G. J. Kapral, R. W. Grosse-Kunstleve, A. J. McCoy, N. W. Moriarty, R. Oeffner, R. J. Read, D. C. Richardson, J. S. Richardson, T. C. Terwilliger and P. H. Zwart, *Acta Crystallogr D Biol Crystallogr*, 2010, **66**, 213-221.
12. A. J. McCoy, R. W. Grosse-Kunstleve, P. D. Adams, M. D. Winn, L. C. Storoni and R. J. Read, *J Appl Crystallogr*, 2007, **40**, 658-674.
13. M. Bhagawati, T. M. E. Terhorst, F. Fusser, S. Hoffmann, T. Pasch, S. Pietrokovski and H. D. Mootz, *Proc Natl Acad Sci U S A*, 2019, **116**, 22164-22172.
14. M. W. Southworth, E. Adam, D. Panne, R. Byer, R. Kautz and F. B. Perler, *Embo J*, 1998, **17**, 918-926.
15. J. Jumper, R. Evans, A. Pritzel, T. Green, M. Figurnov, O. Ronneberger, K. Tunyasuvunakool, R. Bates, A. Zidek, A. Potapenko, A. Bridgland, C. Meyer, S. A. A. Kohl, A. J. Ballard, A. Cowie, B. Romera-Paredes, S. Nikolov, R. Jain, J. Adler, T. Back, S. Petersen, D. Reiman, E. Clancy, M. Zielinski, M. Steinegger, M. Pacholska, T. Berghammer, S. Bodenstein, D. Silver, O. Vinyals, A. W. Senior, K. Kavukcuoglu, P. Kohli and D. Hassabis, *Nature*, 2021, **596**, 583-589.
16. E. F. Pettersen, T. D. Goddard, C. C. Huang, G. S. Couch, D. M. Greenblatt, E. C. Meng and T. E. Ferrin, *J Comput Chem*, 2004, **25**, 1605-1612.
